# The p38/MK2-Driven Exchange between Tristetraprolin and HuR Regulates AU–Rich Element–Dependent Translation

**DOI:** 10.1371/journal.pgen.1002977

**Published:** 2012-09-27

**Authors:** Christopher Tiedje, Natalia Ronkina, Mohammad Tehrani, Sonam Dhamija, Kathrin Laass, Helmut Holtmann, Alexey Kotlyarov, Matthias Gaestel

**Affiliations:** Institute of Biochemistry, Hannover Medical School, Hannover, Germany; Brigham and Women's Hospital Harvard School of Medicine, United States of America

## Abstract

TNF expression of macrophages is under stringent translational control that depends on the p38 MAPK/MK2 pathway and the AU–rich element (ARE) in the TNF mRNA. Here, we elucidate the molecular mechanism of phosphorylation-regulated translation of TNF. We demonstrate that translation of the TNF-precursor at the ER requires expression of the ARE–binding and -stabilizing factor human antigen R (HuR) together with either activity of the p38 MAPK/MK2 pathway or the absence of the ARE-binding and -destabilizing factor tristetraprolin (TTP). We show that phosphorylation of TTP by MK2 decreases its affinity to the ARE, inhibits its ability to replace HuR, and permits HuR-mediated initiation of translation of TNF mRNA. Since translation of TTP's own mRNA is also regulated by this mechanism, an intrinsic feedback control of the inflammatory response is ensured. The phosphorylation-regulated TTP/HuR exchange at target mRNAs provides a reversible switch between unstable/non-translatable and stable/efficiently translated mRNAs.

## Author Summary

For immediate response and better control of gene expression, eukaryotic cells have developed means to specifically regulate the stability and translation of pre-formed mRNA transcripts. This post-transcriptional regulation of gene expression is realized by a variety of mRNA-binding proteins, which target specific mRNA sequence elements in a signal-dependent manner. Here we describe a molecular switch mechanism where the exchange of two mRNA-binding proteins is regulated by stress and inflammatory signals. This switch operates between stabilization and efficient translation of the target mRNA, when the activator protein of translational initiation binds instead of the phosphorylated destabilizing protein, and translational arrest and degradation of the target, when the non-phosphorylated destabilizing protein replaces the activator. This mechanism is specific to the mRNA of the inflammatory cytokine tumor necrosis factor (TNF)-α and the mRNA of its regulator protein TTP and, hence, enables fast inflammatory response and its stringent feedback control.

## Introduction

TNF is a master cytokine of inflammatory signaling of macrophages. Its biosynthesis is tightly controlled to allow rapid secretion but also to avoid delay or leakiness in its down-regulation, which could result in exaggerated or persistent inflammation. The levels of regulation comprise transcription, processing, nuclear export and stability of the TNF mRNA, translation of pro-TNF, and shedding of TNF (reviewed e.g. in [1]). Pro-TNF contains a leader sequence of 79 amino acids (for mouse TNF) and is synthesized as a type II membrane protein [2], [3]. After initiation of ribosomal translation of TNF mRNA, followed by SRP-mediated arrest of ribosomal synthesis of the nascent protein chain and docking to the ER membrane, the C-terminal part of TNF containing the potential cleavage site is synthesized into the lumen of the ER. Subsequently, pro-TNF is transported in an LPS-stimulated manner from the trans Golgi network to the cell surface using tubular carriers that fuse with the recycling endosome [4]. At the cell surface, pro-TNF is cleaved and released by the TNF-converting enzyme TACE/ADAM17 [5].

The p38 MAPK/MK2/3 pathway [6] regulates TNF-biosynthesis mainly at the translational level. Inhibition of this pathway by small molecules, such as SB203580 or SB202190, or deletion of its components, such as the downstream protein kinase MK2, lead to a significant reduction of LPS-induced TNF production of macrophages although the LPS-stimulated increase in TNF mRNA concentration remains almost unaltered and the stability of mature TNF mRNA in these cells is only sightly reduced [7]–[9]. The post-transcriptional regulation of TNF biosynthesis by the p38 pathway depends on the AU-rich element (ARE) in the 3′ non-translated region of TNF mRNA [9], [10]. So far the molecular mechanisms regulating ARE-dependent translation of pro-TNF via phosphorylation are not understood. However, various mRNA- and ARE-binding proteins have been identified as substrates of the p38 MAPK pathway, e.g. hnRNP A0, tristetraprolin (TTP), KSRP and poly(A)-binding protein 1 [11], [12], [13], [14], [15]. The mRNA-ARE and corresponding ARE-binding proteins (ABPs), such as TTP, KSRP, HuR, TIA-1 and AUF1, are mainly held responsible for the regulation of mRNA metabolism governed by exosome-, PARN- and CCR4/Not1-dependent degradation of mRNAs or by their storage in discrete cytoplasmic foci (reviewed in [16] and [17]–[19]). For example, KSRP stimulates the rapid decay of ARE-containing mRNAs and its activity is inhibited via direct phosphorylation by p38 MAPK providing a mechanism of stress-dependent stabilization of ARE-containing mRNAs [13]. In contrast, HuR (ELAV) is a factor of constitutive nuclear and cytoplasmic stabilization of ARE-containing mRNAs [20], [21], which also binds to the ARE of TNF mRNA [22], [23]. However, besides its mRNA stabilizing function, HuR also influences translation of specific mRNAs as measured by the association of these mRNAs to the polysomal fractions of cell lysates separated by density centrifugation. Gene-silencing of HuR blocks polysomal localization of cytochrome C- and nucleolin-mRNA indicating a positive effect of HuR on translation of these mRNAs [24], [25]. In contrast, deletion of HuR increases the long-term shift of TNF mRNA to polysomal fractions after LPS treatment of macrophages [26] and its overexpression leads to a reduction of TNF-mRNA in polysomal fractions [27] indicating also an inhibitory effect of HuR on translation.

The inhibitory effect of TTP in the regulation of TNF production first became obvious by significant cachexia in the TTP knockout mouse [28], which was explained by increased TNF concentrations and a feedback effect of TTP on TNF production by its binding to the ARE and destabilization of the TNF mRNA [29]. Subsequently, TTP could be identified as a destabilizing factor for various ARE-containing mRNAs, including its own mRNA (reviewed in [16]). In LPS-induced TNF biosynthesis TTP is genetically downstream to p38 MAPK and MK2, since its deletion neutralizes the defect in LPS-induced TNF production seen in MK2 knockout mice [30]. MK2 directly phosphorylates TTP [11]. It is proposed that phospho-TTP/mRNA complexes are sequestered by 14-3-3 binding proteins [15] and that phospho-TTP is unable to recruit deadenylases [17], [19] resulting in target mRNA stabilization. Via phosphorylation of SRF, MK2 also contributes to transcriptional activation of the TTP gene [31]. Interestingly, in MK2 knockout and, especially, in MK2/MK3 double knockout (DKO) macrophages a strong reduction of the TTP concentration is observed [32] suggesting a major role of the p38/MK2/3 pathway in the regulation of TTP expression.

The predominant translational regulation of TNF by the p38 MAPK/MK2/3 pathway raises the question of the role played by certain direct substrates of these protein kinases and the mechanisms involved in this translational regulation. Here, we reconstituted MK2-dependent translational regulation of TNF in immortalized MK2/3-deficient mouse macrophages by re-introducing MK2, its catalytic dead mutant or, as a control, GFP alone. We analyzed the requirements of MK2-dependent translation of native TNF mRNA for the presence of ABPs, such as TTP and HuR, and for the mRNA-binding MK2-substrate Ago2. In this analysis, the combination of cytosol/ER-fractionation and subsequent polysome profiling provides additional mechanistic insights into the translational regulation of pro-TNF. The regulatory mechanisms of translation of TNF mRNA elucidated by this approach are also valid for TTP mRNA and contribute to the stringent feedback regulation of the inflammatory response.

## Results

### Reconstitution of MK2-Dependent Post-Transcriptional Control of TNF Biosynthesis in Macrophage Cell Lines

We generated an immortalized macrophage cell line from MK2/MK3 double-deficient mice [32] by expression of v-raf and v-myc in bone marrow derived macrophages (BMDM). For restoration of the “wild type” situation in these macrophages, we subsequently expressed MK2 by stable retroviral transduction using pMMP-MK2-IRES-GFP. As knockout control for this cell line, we used pMMP-IRES-GFP. To differentiate between the effects of catalytic activity of MK2 and the effects of MK2-dependent stabilization of p38 in the binary complex [33], we also rescued the cell line with the catalytic dead mutant of MK2 (pMMP-MK2K79R-IRES-GFP) [31]. The generation of different cell lines by retroviral transduction after initial immortalization excludes the unwanted influence of different random events of immortalization in the cell lines to be compared. After retroviral transduction, cell lines were sorted and selected by preparative FACS for comparable expression of GFP. The level of expression of MK2 in the rescued cell lines was comparable to its level in immortalized wild type BMDM and in RAW264.7 cells (Figure S1).

**Figure 1:**
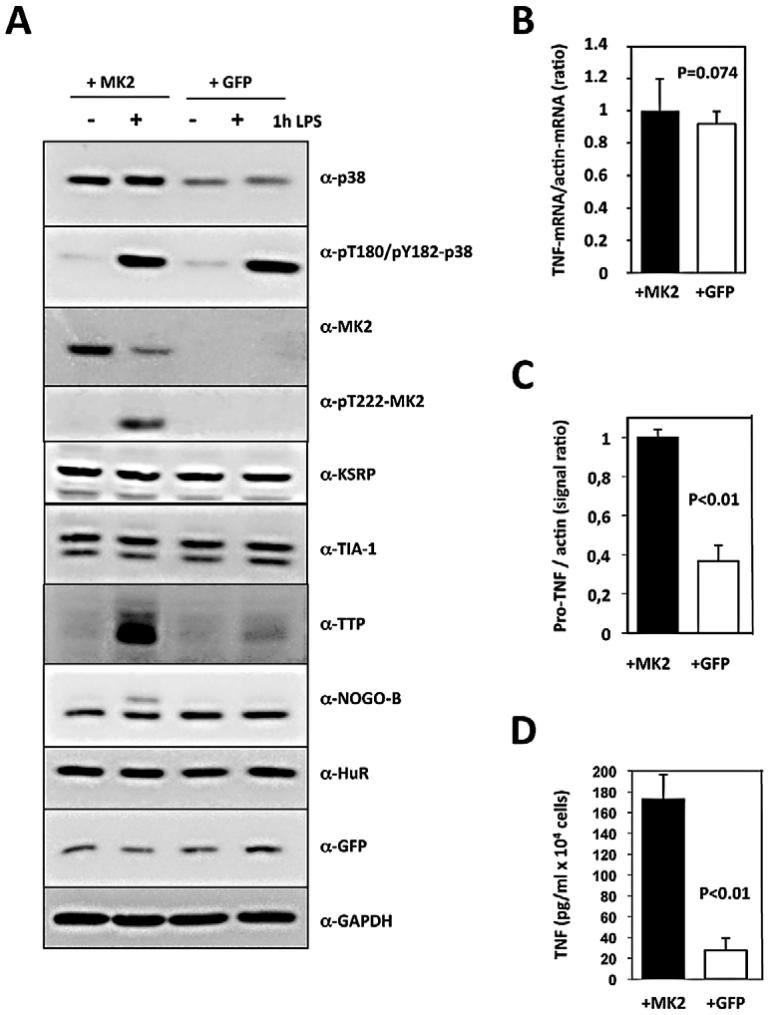
Translational regulation of TNF reconstituted by macrophage cell lines. MK2/3-deficient macrophages were immortalized as described and the immortalized cell line was transduced with retroviral constructs coding for MK2 and GFP (+MK2) or GFP alone (+GFP), respectively. A) Western blot analysis of LPS (1 µg/ml)-induced protein expression and phosphorylation of the protein kinases p38 and MK2. Compared with MK2-rescued “wild type” cells, GFP-transduced “knockout” cells display absence of MK2, NOGO-B phosphorylation and a reduced TTP concentration. B) Relative TNF mRNA level in MK2- and GFP-rescued cells 1 h after LPS treatment as determined by RT-qPCR of TNF and ß-actin mRNA does not differ significantly. C) Comparison of pro-TNF protein expression between MK2- rescued and GFP-transduced cells 1 h after LPS-treatment. Pro-TNF level was calculated from a quantitative evaluation of the chemoluminescence of specific Western blot signals of total cell lysates (cf. Figure 3D). D) Comparison of the amount of secreted TNF between MK2- rescued and GFP-transduced cells measured by ELISA 1 h after LPS stimulation. P values of the two-tailed student's t-test are given.

We compared basal and LPS-dependent expression and phosphorylation of the central components of the pathway (p38, MK2) and of relevant substrates of MK2/3 (TTP, NOGO-B) in the cell lines (Figure 1A and Figure S2A). As controls, we also analyzed expression of HuR, TIA-1, KSRP, GFP and GAPDH. While these controls showed comparable expression in both cell lines and were not induced by LPS, we detected strong induction of p38 activity by LPS in both cell lines. Although decreased stabilization of p38 protein by the lack of MK2/3 leads to reduced p38 concentrations in GFP-transduced MK2/3-deficient cells, the activity of p38, detected by the antibody which only detects the dual phosphorylation in the activation loop, was similar in MK2-rescued and GFP-transduced cells. Obviously, increased p38 activation compensates for its lower expression not only in neurons [34], but also in macrophages. As expected, MK2 is only detected and activated by LPS-treatment in MK2-rescued cells. Interestingly, there is a rapid induction of the MK2/3 substrate TTP by LPS-treatment, qualifying TTP as an immediate early gene. This LPS-induced expression of TTP is strongly reduced in GFP-transduced cells, a fact already known from MK2- and MK2/3-deficient primary cells [32]. In addition, a lack of phosphorylation of the ER membrane resident MK2 substrate NOGO-B, which is phosphorylated in its cytoplasmic domain [35], is detected in GFP-transduced compared to MK2-rescued cells based on the loss of the slower migrating phosphorylated isoform of NOGO-B.

Since the kinetics of p38/MK2 activation and TNF production in macrophages is fast (MK2 activity peaks after 20 min, maximum of TNF production is reached after about 60 min), we determined TNF-mRNA and -protein concentration 1 h after LPS-treatment. The relative intracellular TNF mRNA concentration as represented by the TNF mRNA/actin mRNA ratio and the length of the polyA-tail of TNF-mRNA do not significantly differ between MK2-rescued and GFP-transduced cells (Figure 1B and Figure S3). In contrast, both pro-TNF protein and secreted TNF are significantly increased in MK2-rescued cells compared to the GFP-transduced control (Figure 1C, 1D). Thus, the translational control of TNF biosynthesis by MK2 is clearly reflected in this cellular system. The catalytic activity of MK2 is necessary for this control, since rescue of the macrophages with the kinase-dead mutant MK2-K79R does not release the translational repression of TNF (Figure S2).

### MK2-Dependent Translation of TNF mRNA Monitored by ER–Localization and Ribosome Occupancy

**Figure 2:**
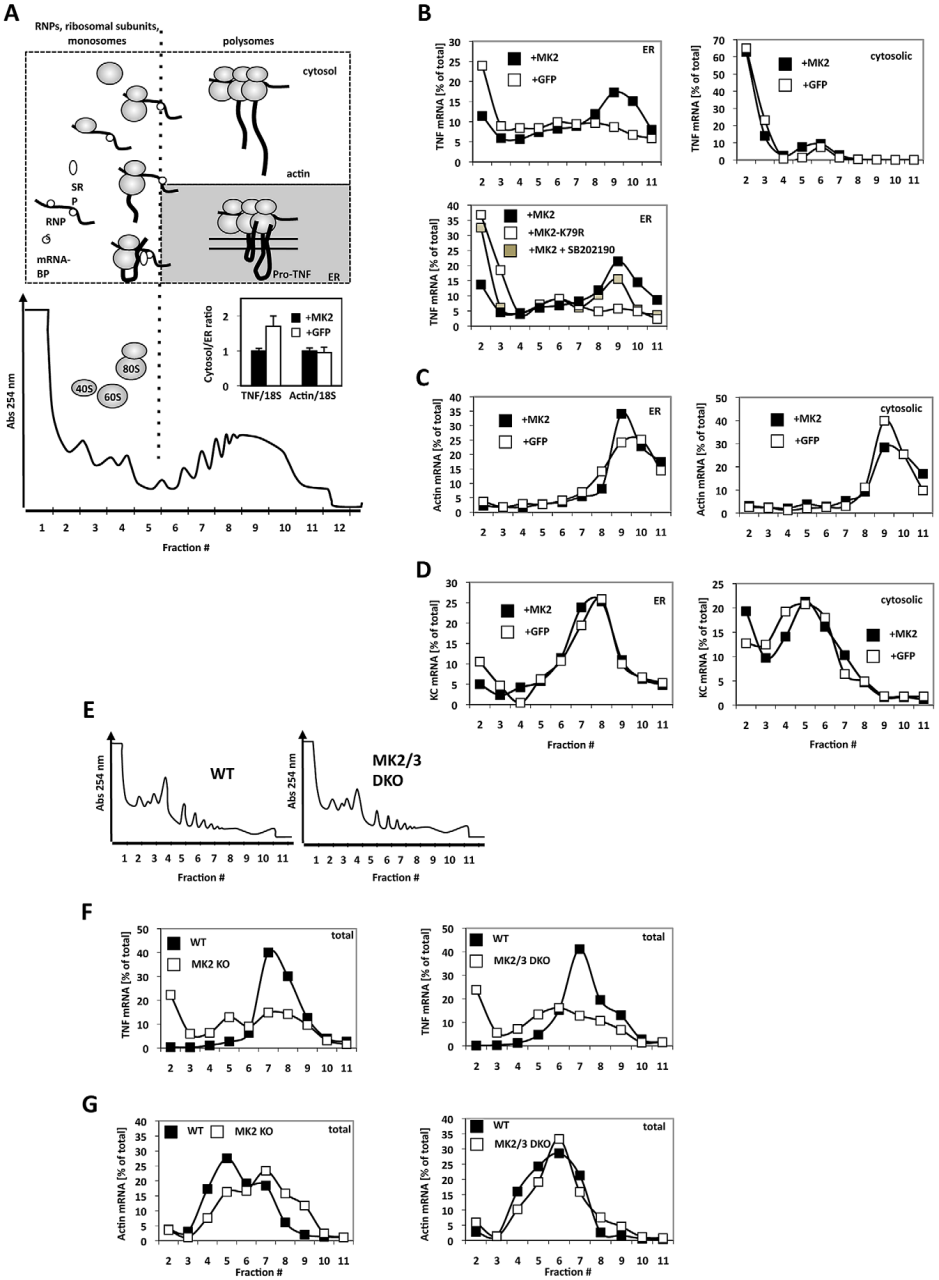
MK2-dependent translational control of TNF mRNA detected by ribosomal profiling after ER/cytosol cell fractionation of MK2-rescued and GFP-transduced macrophages and of wild type, MK2-deficient, and MK2/MK3 double-deficient primary BMDM. A) Polysome profile and fractions (1–12) within the profile. A schematic representation of the position of RNPs, ribosomal subunits, monosomes and polysomes is given above. Insert: TNF and ß-actin mRNA distibution (cytosol/ER ratio) after LPS-treatment of MK2-rescued (+MK2) or GFP-transduced (+GFP) macrophages. The difference in the TNF/18S is significant with p = 0.007. B) Distribution of TNF mRNA in the ER and cytosolic polysome profiles of MK2-rescued and GFP-transduced cells 1 h after LPS stimulation. An MK2-dependent redistribution of TNF mRNA to polysomal ER fractions (peaking in fraction 9) is observed. Rescue with the catalytic dead mutant MK2 K79R and block of MK2 activation by the p38 inhibitor SB202190 (MK2+SB202190) leads to a loss of the redistribution. C) MK2-independent distribution of ß-actin mRNA in the polysome profiles. D) MK2-independent distribution of the mRNA of the secreted cytokine KC/Cxcl1. E) Polysome profiles of total lysates of wild type, MK2-deficient and MK2/MK3 double-deficient primary BMDM. F,G) Distribution of TNF mRNA (F) and actin mRNA (G) in the polysome profile of WT, MK2-deficient and MK2/3-deficient primary macrophages.

To monitor ribosome occupancy of TNF mRNA in these cells as a measure of translational initiation and elongation, we applied density gradient centrifugation to distinguish between TNF mRNPs, TNF mRNA containing monosomes and polysomes. Furthermore, since pro-TNF is synthesized as a type II membrane protein by ER-directed translation, we decided to combine cytosol/ER-fractionation by saponin treatment, which was modified after [36], with subsequent density centrifugation. A typical fractionation is shown in Figure 2A. While mRNPs, ribosomal subunits, scanning ribosomal subunit, initiated translation and stalled ribosomes with signal recognition particle (SRP) bound to the nascent peptide chain before docking to the ER membrane should distribute between fractions 1–4, polyribosomes are expected in fraction 6 and above. Polyribosomes containing mRNAs of secreted or membrane proteins, such as pro-TNF, are expected in the polyribosomal fractions of the ER subfraction, while polyribosomes containing mRNAs of cytosolic proteins together with all monosomes including SRP-stalled monosomes with nascent proteins are expected in the cytosolic subfraction. The overall distribution of TNF mRNA after LPS-stimulation differs between MK2-rescued and GFP-transduced cells as seen for the cytoplasm/ER ratio, which is significantly lower in MK2-rescued cells (Insert in Figure 2A and Figure S4 for absolute mRNA levels), while the distribution of ß-actin mRNA is not significantly different. Thus MK2 is required for preferential ER localization of TNF mRNA. We then performed polysome profile analyses of specific mRNAs. To ensure biological significance, we always performed at least two independent experiments comprising separate LPS-stimulation of cells, density gradient fractionation of cell lysates and qRT-PCR detection of specific mRNAs. Data were only considered significant in the instances where the biological repeats yielded the same qualitative results. The repeats of key experiments are displayed in Figure S5. As seen in the polysome profiles for TNF mRNA (Figure 2B), its cytosolic population occurs mostly in the free mRNP fraction. Only a low concentration of monosomal complexes of TNF mRNA is detected in the cytosolic fraction of MK2-transduced cells and, to a slightly lower degree, also in GFP-transduced cells. In the ER fraction, a clear peak of larger polysomal complexes of TNF mRNA (fractions 9–10) is seen in the presence of MK2 and this peak is completely missing in the absence of MK2 in cells transduced with GFP only. In parallel, the free mRNP signal is decreased for TNF mRNA in the presence of MK2. The existence of the larger polysomal complexes of TNF mRNA depends on catalytic activity of p38 and MK2, since the peak corresponding to larger polysomal complexes is reduced after treatment of the cells with the p38 inhibitor SB202190 and completely disappears for cells expressing the catalytic dead MK2 mutant instead of wild type MK2, respectively (Figure 2B). As a control, efficient translation of actin mRNA in ER and cytosol fractions is detectable for both MK2-rescued and GFP-transduced cells (Figure 2C). The finding that ß-actin mRNA coding for a cytosolic protein is also translated at the ER indicates the fact that not all proteins synthesized at the ER are secreted or integral membrane proteins [37] and that ER-associated mRNAs serve a global role also for translation of cytosolic proteins [38]. As further control, we monitored the mRNA distribution of the secreted chemokine KC (Cxcl1), which is also synthesized as pro-protein at the ER and which is known to be regulated by MK2 at the level of mRNA stability, but not at the level of translation [32]. KC mRNA distribution is independent of the presence of MK2 (Figure 2D). Efficient translation of this mRNA proceeds mainly at the ER. However, KC mRNA peaks in earlier polysome fractions (around 7–8) compared to TNF mRNA (peak at fractions 9–10), since the ORF of KC mRNA is 291 nucleotides only, compared to 708 nucleotides of TNF mRNA, allowing only a smaller number of ribosomes to elongate at the same mRNA molecule. In contrast to ß-actin mRNA, KC mRNA in the cytosolic fraction is mainly detected in monosomes. These monosomes probably represent translationally initiated monosomes with the SRP-arrested nascent protein chain. In cytosolic fractions the amount of ribosome-free KC mRNPs is comparable to the amount of KC mRNA in monosomes. In contrast, the amount of ribosome-free TNF mRNPs is significantly higher than the amount of monosomal TNF mRNA, indicating that initiation is the critical step for TNF mRNA translation. Taken together, this fractionation analysis clearly demonstrates a specific role for the catalytic activity of the protein kinase MK2 in the regulation of ER-directed translation of pro-TNF in macrophages.

To demonstrate that the cell lines generated reflect the situation in primary BMDMs we extended the analysis of LPS-induced MK2-dependent translation of TNF mRNA to wild-type, MK2-deficient and MK2/3 double-deficient primary BMDM. There are no qualitative differences in the overall polysome profiles of wild type and MK2/MK3 double-deficient BMDM (Figure 2E). However, after 1 h of LPS-stimulation TNF mRNA is detected in a clear polysomal peak for wild type cells only, while in MK2- and MK2/MK3-deficient cells this peak is missing (Figure 2F). As control, distribution of ß-actin mRNA in the polysome profile does not show clear differences between wild type and knockout BMDM (Figure 2G).

### Analysis of the Distribution of Specific Proteins in the Monosome/Polysome Gradient and in the Cytosolic/ER Fractions

**Figure 3:**
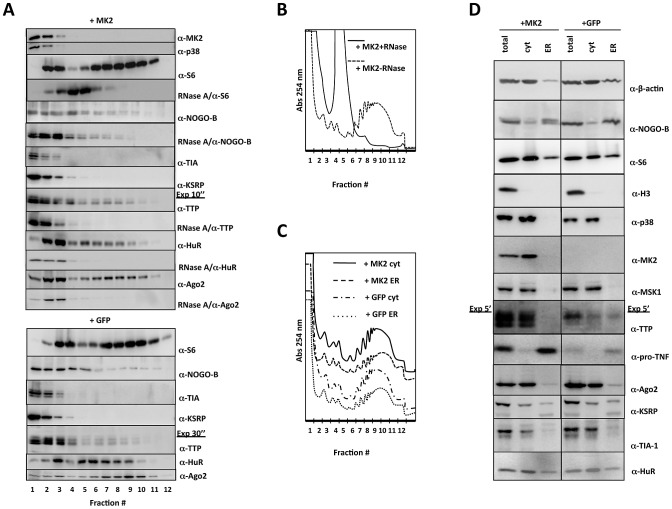
Western blot analysis of protein distribution in polysome profiling and ER/cytosolic sub-fractionation. A) Distribution of relevant proteins in the polysome profile (fractions 1–12) of MK2-rescued (+MK2) and GFP-transduced (+GFP) cells. Where indicated an RNaseA treatment was carried out before polysome profiling to monitor requirement of RNA-binding for the distribution. B) Polysome profile of LPS-treated MK2-rescued macrophages before and after RNaseA treatment. C) Polysome profiles of cytosolic and ER subfractions of LPS-treated MK2-rescued and GFP-transduced macrophages. D) Distribution of relevant proteins in the ER and cytosolic (cyt) fraction of MK2-rescued (+MK2) and GFP-transduced (+GFP) cells. Total lysate before fractionation (total) is shown as control.

We postulated that proteins involved in the p38/MK2-dependent regulation of translation should exist in specific fractions of the monosome/polysome profile or the cytosol/ER fractions of LPS-treated macrophages depending on the presence of MK2. Therefore, we analyzed the relative concentration of various candidate proteins in the different fractions of lysates of LPS-stimulated MK2-rescued or GFP-transduced macrophages (Figure 3). In the density centrifugation of total cell lysates (Figure 3A), MK2 and p38 - as freely diffusible small proteins - are mainly present in the ribosome-free fraction, which contains also mRNPs. The small ribosomal protein S6, which was used to monitor ribosome distribution, is present in monosomal and polysomal fractions independent of the presence of MK2, indicating that there is no general effect of MK2 on translation (cf. also Figure 2C, 2D). Transcript regions free of bound ribosomes are cleaved by RNaseA treatment resulting in destruction of polyribosomes (Figure 3B) and in S6 being shifted to monosomes (Figure 3A).

The MK2 substrate NOGO-B is present in the ribosome-free mRNP, the monosomal and polysomal fractions, indicating that monosomes and polysomes may dock to the ER. A double band for NOGO-B, characteristic of the slower migrating phosphorylated and faster migrating non-phosphorylated isoform, is only seen in MK2-rescued cells in all fractions indicating that NOGO-B is a specific substrate for MK2 also in macrophages (cf. Figure 1A) and that phosphorylation by MK2 does not change its overall distribution in the gradient. Since remaining ER structures cannot be degraded by RNase A, NOGO-B distribution is not completely changed after RNase treatment and seems not to be a ribosome-associated protein.

The distribution of various mRNA-binding proteins (TIA-1, KSRP, TTP, HuR, and Ago2) was analyzed. RNA binding of TTP, HuR and Ago2 is necessary for their distribution in the gradient, since RNase A pre-treatment leads to almost complete disappearance of these proteins from the gradient (Figure 3A). As for NOGO-B, the lack of phosphorylation of TTP by MK2 in the GFP-transduced macrophages does not significantly change the overall distribution of TTP in the gradient. Remarkably, only HuR and Ago2 show a clear two peak-distribution corresponding to the peaks of monosome and polysome fractions as represented by the S6 distribution. This observation strengthens the notion that these proteins may control ribosomal translation. There is no significant MK2-dependent redistribution detected for the proteins analyzed. This is not unexpected, since TNF mRNA and other ARE-containing mRNAs are only a minor part of the total cellular mRNAs analyzed in this overall fractionation.

We also compared the distribution of selected proteins between the cytosolic and ER fractions in dependence on the presence of MK2. There are no qualitative differences in the overall polysome profiles of MK2-rescued and GFP-transduced cells or of the ER or cytosol fractions detected after 1 h LPS-stimulation as measured by absorbance at 254 nm (Figure 3C). However, ß-actin is mainly present in the cytosolic fraction, while the major portion of NOGO-B is detected in the ER fraction (Figure 3D). The ribosomal protein S6 is present in both fractions representing cytosolic and ER-directed translation. The nuclear histone protein H3 is detectable in the total extract but not in the cytosolic or ER fractions, indicating that the fractionation indeed excludes nuclei. The distribution of ß-actin, NOGO-B, S6 and H3 is independent of the presence of MK2. p38 MAPK, MK2, and another protein kinase downstream to p38, Msk1 [39], are detected exclusively in the cytosolic fraction. Only in MK2-rescued macrophages, NOGO-B displays the double band for the phosphorylated and non-phosphorylated isoforms in the ER-fraction, indicating that NOGO-B can serve as a substrate for MK2 although both proteins are detected in different fractions. Most interestingly, TTP is detectable in the cytosol and ER of GFP-transduced cells, but shows a clear exclusion from the ER fraction in MK2-rescued macrophages, although expressed to a higher total concentration in these cells. Such complete exclusion is not seen for the other mRNA-binding proteins tested (Ago2, KSRP, TIA-1, and HuR). The MK2-dependent exclusion of TTP from the ER fraction containing ER-bound polysomes, also loaded with TNF mRNA (cf. Figure 2B), indicates a possible inhibitory role of TTP in translational regulation, which can be neutralized by MK2. In accordance with its synthesis as a type II-membrane protein, pro-TNF is almost exclusively detected in the ER franction and its level is significantly reduced in the GFP-transduced cells (Figure 3D, cf. Figure 1C)

### Knockdown of TTP Specifically Increases TNF Translation in an MK2-Independent Manner

**Figure 4:**
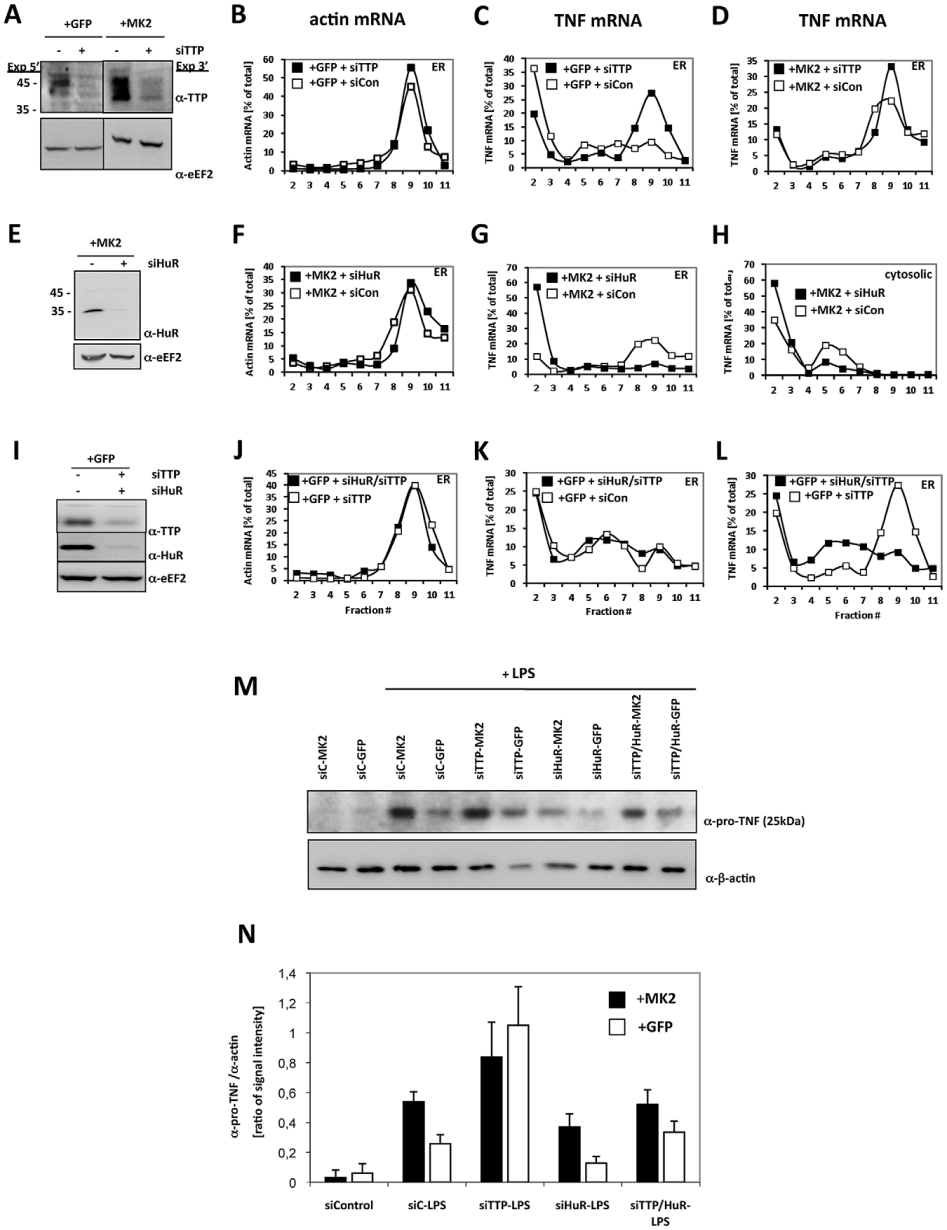
TTP and HuR oppositely regulate translation of TNF mRNA. A) Knockdown of TTP in MK2-rescued and GFP-transduced macrophages, corresponding Western blot analysis of knockdown efficiency (−siTTP = siCon−scrambled siRNA, +siTTP−TTP specific siRNA), B) polysome profiles for ß-actin mRNA of the ER fraction of LPS-treated GFP-rescued cells and for TNF mRNA of the ER fraction of LPS-stimulated GFP-transduced (C) and MK2-rescued cells treated with siTTP or scrambled control (+siCon) (D). E) Knockdown of HuR in MK2-rescued macrophages, corresponding Western blot analysis of knockdown efficiency (−siHuR = siCon−scrambled siRNA, +siHuR−HuR specific siRNA), polysome profiles for ß-actin mRNA of the ER fraction of LPS-stimulated MK2-rescued cells (F) and for TNF mRNA of the ER (G) and cytosolic fractions (H) of LPS-stimulated MK2-rescued cells treated with siHuR or scrambled control (+siCon). I) Parallel knockdown of TTP and HuR in GFP-transduced macrophages, corresponding Western blot analysis of knockdown efficiency (−siHuR, −siTTP = siCon−scrambled siRNA, +siHuR, +siTTP−HuR and TTP specific siRNAs), J) polysome profiles for ß-actin mRNA of the ER fraction of LPS-stimulated GFP-transduced cells and for TNF mRNA of the ER fraction of LPS-stimulated GFP-transduced cells treated with siHuR/siTTP, siTTP alone or scrambled control (+siCon) (K, L). M,N) pro-TNF production of the macrophage cell lines treated with siRNA for knockdown of TTP, HuR, TTP/HuR determined in duplicate 4 h after LPS stimulation. M) Representative Western blots for pro-TNF quantification. The ß-actin signal was used for normalization of differences in loading and transfer efficiency. siC = siCon−scrambled siRNA. N) Quantitative representation of the Western blot signal for pro-TNF. The mean and SD of the ratio between pro-TNF and ß-actin signal of two independent experiments with two different expositions and quantifications each are shown.

The MK2-dependent absence of TTP in the ER fraction, where TNF mRNA is actively translated, and the fact that TTP is a substrate for MK2 [11] at least indirectly suggest an MK2-dependent role of TTP in repression of TNF translation. This notion is also in agreement with the observation that genetic deletion of TTP increases TNF production and renders it MK2-independent [30]. To prove this hypothesis, we performed TTP knockdown (Figure 4A) and analyzed ER-directed pro-TNF translation. As seen in Figure 4B, TTP knockdown does not influence translation of ß-actin mRNA. Interestingly, TTP knockdown specifically stimulates a significant shift of TNF mRNA into ER-bound polysome fractions in LPS-treated GFP-transduced macrophages (Figure 4C). Since in LPS-treated MK2-rescued macrophages TNF mRNA is already detected in the polysomal fractions, the amount of polysomal TNF mRNA is only slightly increased further by the knockdown of TTP (Figure 4D). These results clearly identify TTP as a specific repressor of TNF translation, which acts downstream of MK2.

### HuR Is Necessary for MK2-Dependent and -Independent Translation of Pro-TNF

Since the distribution of HuR in density centrifugation parallels ribosomal distribution (Figure 3A) and since it is known that HuR also binds to the ARE of native TNF-mRNA [23], we analyzed the effect of knockdown of HuR on the translation in MK2-rescued macrophages. siHuR knockdown is efficient and does not significantly influence translation of ß-actin mRNA (Figure 4E, 4F). Remarkably, upon HuR knockdown TNF mRNA is excluded from polysomal ER-bound fractions (Figure 4G), reduced in monosomal cytosolic fractions and increased in the mRNP fractions (Figure 4H). This indicates that HuR is necessary for the translation and specifically involved in translational initiation of TNF mRNA.

We then asked whether the de-repression of translation seen upon TTP knockdown also depends on the presence of HuR. We performed double siRNA knockdown of TTP and HuR in LPS-treated GFP-transduced macrophages (Figure 4I). Knockdown is efficient for both proteins. As for the TTP knockdown alone, the TTP/HuR double knockdown does not influence translation of ß-actin mRNA (Figure 4J). In contrast, the polysome localization of TNF mRNA induced by TTP knockdown (cf. Figure 4C) is not observed in the TTP/HuR double knockdown (Figure 4K, 4L) indicating that HuR is necessary for both types of de-repression, whether caused by the reduction or phosphorylation of TTP. Hence, in contrast to TIA-1, which acts as constitutive repressor [40], HuR is a constitutive activator of TNF translation in these cells.

### Ago2 Does Not Affect ER-Directed Translation of Pro-TNF

Similar to HuR, Ago2 also parallels ribosomal distribution (Figure 3A). Ago2 has been described to be essential for TTP- and miR16-dependent TNF mRNA degradation [41] and for translational regulation of TNF mRNA upon serum starvation [42], [43]. Furthermore, Ago2 is also a substrate for MK2 [44]. Therefore, we analyzed the role of Ago2 in MK2-dependent translational control of TNF mRNA by knockdown experiment. Although Ago2 itself is a central element of the mechanism of siRNA-mediated regulation of specific mRNAs, its knockdown is quite efficient in MK2-rescued and GFP-transfected cells (Figure S6). However, knockdown of Ago2 does not significantly alter TNF mRNA distribution in MK2-rescued or GFP-transduced macrophages (Figure S6, lower panels) indicating that Ago2 is not regulating LPS-induced translation of TNF mRNA under these conditions.

### Pro-TNF Production Correlates with the Amount of TNF mRNA in Polysomes upon Down-Regulation of HuR and TTP

We asked whether the changes in the presence of TNF mRNA in the polysomal fractions seen for the down-regulation of the different components in MK2-rescued and GFP-transduced macrophages are also reflected by changes in pro-TNF biosynthesis of these cells. For this reason, pro-TNF levels before and 4 h after LPS stimulation were semi-quantitatively determined by Western blot analysis. For two independent experiments Western blot signals were quantified and normalized by the ß-actin signal (Figure 4M, 4N). Before LPS stimulation almost no pro-TNF signal was detectable (Figure 4M), indicating that the pro-TNF detected after LPS-treatment represents newly synthesized protein. After LPS-stimulation the amount of newly synthesized pro-TNF is significantly higher in MK2-rescued compared to the GFP-transduced cells as also seen in Figure 1C and Figure 3D. For MK2-rescued cells the knockdown of TTP, which caused a slight increase in the TNF mRNA polysomal fraction (Figure 4A), also lead to a further small increase in pro-TNF synthesis (Figure 4N). Knockdown of TTP in the GFP-transduced macrophages resulted in a strong increase in pro-TNF level corresponding to the strong shift of TNF mRNA to the polysomal fraction (cf. Figure 4C). Consistently, knockdown of HuR significantly inhibited pro-TNF synthesis in MK2-rescued cells reflecting the decrease in the polysomal amount of TNF mRNA detected under this condition (cf. Figure 4G). Most importantly, parallel knockdown of TTP and HuR resulted in a strongly decreased pro-TNF synthesis compared to the single TTP knockdown reflecting the differences in polysomal TNF mRNA seen in Figure 4L. Taken together, the strict correlation between the abundance of TNF mRNA in the polysomal fraction and the production of pro-TNF after LPS treatment confirms the notions that the TNF-mRNA detected in the polysomal fractions is translationally active and that translation of pro-TNF is regulated by TTP and HuR.

### TTP/HuR Exchange at the TNF–ARE *In Vitro* Is Regulated by MK2

**Figure 5:**
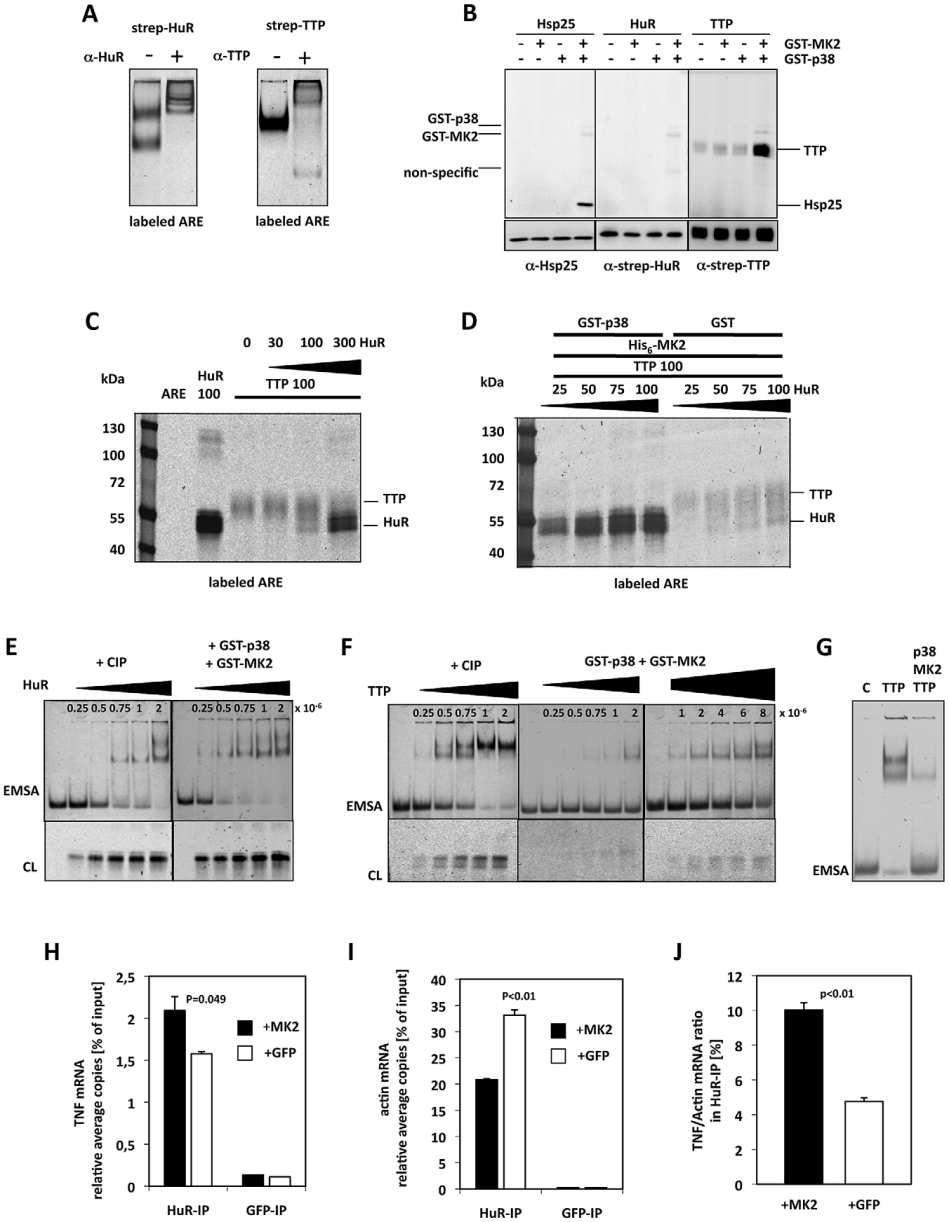
MK2-dependent competition in TNF–ARE binding of HuR and TTP *in vitro* and in LPS-stimulated macrophages. A) Electrophoretic mobility shift assay (EMSA) of an ARE mRNA fragment with purified HuR and TTP and supershift of the specific bands by specific antibodies, respectively. B) *In vitro* phosphorylation of TTP by activated MK2 in the presence of gamma-^33^P-ATP. Recombinant GST-p38 was used to activate recombinant GST-MK2, which then phosphorylates the bona-fide substrate Hsp25 (as control) and TTP. Autophosphorylation of GST-p38 and GST-MK2 is also detected. C) Competition in binding of TTP and HuR to the ARE probe in the absence of MK2 activity (protein amounts in ng). Infrared images of proteins cross-linked to the ARE probe and separated in SDS-Page are shown. D) p38/MK2-dependence of the equilibrium of binding of TTP and HuR to the ARE. E,F) Titrations for the estimation of the ARE-binding affinity (represented by approximate K_d_ values) for CIP-dephosphorylated and p38/MK2 phosphorylated HuR (E) and TTP (F). Samples were analyzed by EMSA and by cross-linking (CL). E) HuR concentrations between 0.25–2×10^−6^ (as indicated) were applied to the binding assays. F) TTP concentrations between 0.25–8×10^−6^ were applied to the binding assays. G) Comparison of ARE-binding between identical concentrations (4×10^−6^ M) of dephospho- (TTP) and phospho-TTP (p38, MK2, TTP) by EMSA. H–J) HuR-RNA-IP using lysates of MK2-rescued and GFP-transfected macrophages after 1 h of LPS-stimulation. H) TNF mRNA in the HuR-IP was quantified in two independent samples. GFP-RNA-IP was analyzed as negative control. I) ß-actin mRNA in the HuR- and GFP-IP. J) TNF mRNA/ß-actin mRNA ratio in the HuR-IP of MK2-rescued and GFP-transduced macrophage cell lines. P values of the two-tailed student's t-test are given.

Since TTP and HuR are both able to bind to the ARE of TNF-mRNA [23], [29] and since HuR is necessary for the release of the translation block after phosphorylation or knockdown of TTP (Figure 4L), we were interested in whether both proteins compete in ARE-binding and whether this competition is controlled by phosphorylation of TTP by MK2/3. To analyze this scenario *in vitro*, we first expressed strep-tagged HuR and TTP in HEK293T cells and purified these proteins by binding to strep-tactin beads. We subjected different amounts of purified strep-HuR and -TTP to Western blotting and detected the proteins using enzyme-coupled strep-tactin. While strep-HuR is detected in a single band of approximately 40 kDa, strep-TTP is seen in a more diffuse group of bands around 50 kDa, which probably arises from multiple PTMs [45] introduced into TTP also in the HEK 293T cells (Figure S7A). To prove ARE-binding activity of the purified proteins, we performed EMSA using a TNF mRNA ARE-derived RNA probe containing six overlapping AUUUA pentamers labeled with the infrared dye DY-681, which can be detected by imaging (Figure 5A). In EMSA, HuR displays the typical two band pattern [23] while TTP retards the ARE probe in one band, as already known [46]. Specific antibodies against HuR and TTP lead to supershifts respectively demonstrating the identity and specificity of the complexes (Figure 5A). In an *in vitro* kinase assay, TTP but not HuR is further phosphorylated by MK2 in the presence of its activator p38 (Figure 5B). The observation that strep-HuR is not phosphorylated by p38 in this experiment is in contrast to the published finding that GST-HuR is phosphorylated by p38 *in vitro* [47] and indicates that the same site is already phosphorylated by Chk2 during overexpression in the HEK293 cells as already described for HeLa cells [48].

We then incubated the purified proteins and mixtures thereof with the DY-681-labeled ARE mRNA, stabilized protein-RNA-complexes by UV cross-linking and visualized ARE-probe bound to protein after SDS-PAGE by infra-red imaging (Figure 5C). Although cross-linking of the ARE-probe to HuR seems more efficient, binding of the probe to both proteins can be detected. In the mixture both proteins show competitive binding to the probe with similar binding in comparable concentrations and a significant displacement of the competitor at about threefold molar excess of the other protein (Figure 5C). Competitive binding of TTP and HuR to the ARE was then analyzed in the presence of active MK2 (Figure 5D). Remarkably, p38/MK2 activity strongly and robustly shifts the binding equilibrium towards HuR in this assay. Even in the presence of an about four-fold molar excess of TTP (100 ng TTP vs. 25 ng HuR), almost exclusive binding of HuR to the ARE is detected in the presence of p38 and MK2. We also tested whether MK2 or p38 alone are able to shift the binding towards HuR (Figure S7B). It became clear that only MK2 together with its activator p38 is able to initiate the TTP-HuR-exchange at the ARE-probe, indicating the necessity of catalytically active MK2 in the assay. We then analyzed the ARE binding affinity of HuR and TTP, respectively, phosphorylated by p38/MK2 activity by EMSA and cross-linking experiments using CIP-dephosphorylated protein as control. By titrating increasing concentrations of these proteins (HuR: 2.5×10^−7^ M–2×10^−6^ M) with the same concentration of the ARE probe (7.5×10^−14^ M) approximate K_d_ values were determined as described in [49]. In line with being no substrate for MK2 (Figure 5B), no significant change in binding affinity of HuR to ARE (kd 5.0–7.5×10^−7^) could be observed in the presence of catalytically active MK2 (Figure 5E). In contrast, catalytic activity of p38/MK2 lead to a clear reduction of the affinity of TTP to ARE represented by a shift in the approximate K_d_ from 5×10^−7^ for the non-phosphorylated protein to 6–8×10^−6^ in the presence of active MK2 (Figure 5F). At a fixed TTP concentration of 4×10^−6^ M significant changes in ARE-binding depending on p38/MK2 phosphorylation could be demonstrated by EMSA (Figure 5G). The reduction in affinity was dependent on phosphorylation of TTP by MK2, since the MK2 phosphorylation site mutant TTP-AA bound with the same affinity to the ARE like the CIP-treated TTP (not shown) and did not show altered affinity in the presence of p38/MK2 (Figure S7D). Furthermore, the binding equilibrium between HuR and the TTP single or double mutants was not changed in the presence of p38/MK2 (Figure S7E). The fact that phosphorylation by MK2 weakens the affinity of TTP for ARE while HuR affinity remains nearly unchanged qualitatively explains that the competitive binding equilibrium between TTP and HuR to the ARE is shifted towards HuR in the presence of MK2.

### MK2-Dependence of Binding of HuR to TNF–mRNA in LPS-Stimulated Macrophages

To examine whether the MK2-dependent shift of the ARE-binding equilibrium between TTP and HuR is relevant *in vivo* in LPS-stimulated macrophages, we monitored the binding of endogenous HuR to TNF mRNA by immunoprecipitation of endogenous HuR (RNA-IP). MK2-rescued and GFP-transduced macrophages were stimulated for 1 h with LPS and the IP was carried out using HuR- and, as negative control, GFP-antibodies. TNF mRNA in the IPs was quantified by qRT-PCR. Compared to the GFP-IP, specific accumulation of TNF mRNA in the HuR-IP is detected (Figure 5H). Since it is known that HuR constitutively binds to an U-rich element in the 3′UTR of ß-actin mRNA [50] and since ß-actin mRNA is not induced by LPS, we quantified ß-actin mRNA in the HuR-IP to monitor the efficacy and further specificity of the IP (Figure 5I). When normalizing the HuR-IP by ß-actin mRNA, a significantly increased TNF mRNA/ß-actin mRNA ratio is observed in LPS-treated MK2-rescued macrophages compared to the GFP-transduced cells (Figure 5J). This finding suggests that an MK2-dependent shift of the binding equilibrium between HuR and TTP at the ARE of TNF mRNA might also occur *in vivo*.

We could not obtain complementary data for TTP binding to TNF mRNA, since the efficacy of TTP-IP using the available antibodies is low. In addition and more importantly, the strong difference in the expression level of TTP between MK2-rescued and GFP-transfected cells (Figure 1A, Figure 3D) leads to large variations in IP-efficiency which make a comparison between these cell lines impossible.

### TTP Expression Is Translationally Controlled Similar to TNF

**Figure 6:**
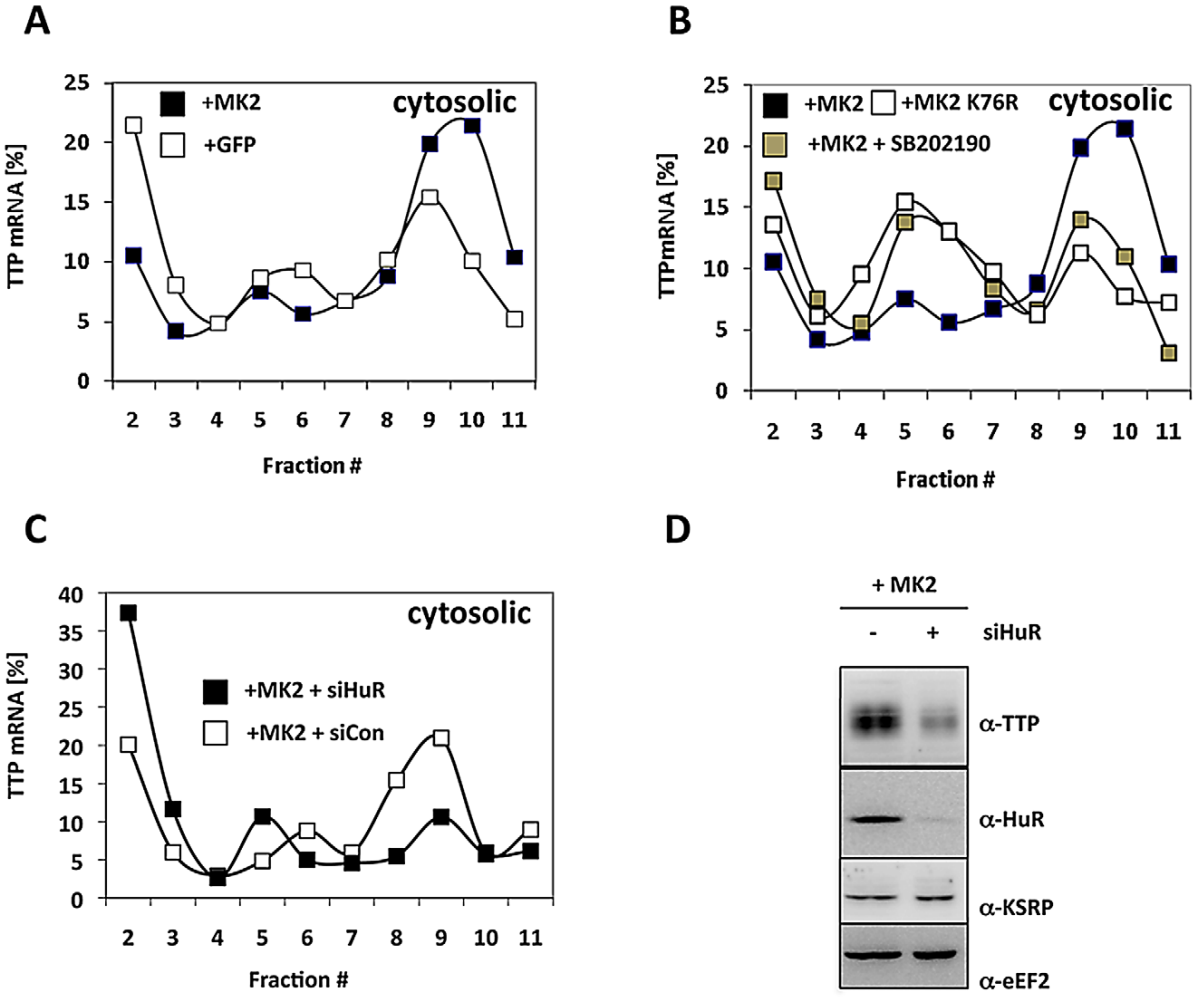
MK2- and HuR–dependent translational regulation of TTP mRNA. A) Cytosolic polysomal profiles for TTP mRNA of LPS-stimulated MK2-rescued and GFP-transduced macrophages. B) Cytosolic polysomal profiles of TTP mRNA depend on catalytic activity of p38 and MK2. C) siRNA mediated knockdown of HuR (MK2+siHuR) inhibits MK2-dependent translation of TTP mRNA. D) Knockdown of HuR decreases LPS-induced TTP expression. Western blots of macrophage lysates prepared 4 h after LPS-induction.

Since the mechanism elucidated regulates initiation of translation before docking to the ER, we were interested in whether synthesis of cytoplasmic proteins is regulated in the same manner. TTP itself is a cytoplasmic protein and it is known that TTP also binds to its own mRNA, which contains a 3′ UTR ARE of three clustered AUUUA pentamers [51]. Therefore, we analyzed translation of TTP mRNA in the cytosolic fraction for its dependence on MK2 catalytic activity and on the presence of the TTP antagonist HuR (Figure 6). The content of TTP mRNA in polysomal fractions is significantly increased in MK2-rescued macrophages when compared to GFP-transduced cells (Figure 6A). The p38 inhibitor blocks this increase and the catalytically dead mutant of MK2 is not able to increase TTP translation (Figure 6B). Both treatments increase the concentration of TTP mRNA in monosomes. These findings indicate that catalytic activity of MK2 also stimulates translation of TTP mRNA in the cytosol. Interestingly, knockdown of HuR strongly inhibits MK2-mediated translational stimulation of TTP mRNA and leads to the accumulation of TTP mRNA in mRNPs (Figure 6C). This inhibition is accompanied by a decrease in TTP biosynthesis as detected by Western blot 4 h after LPS-stimulation (Figure 6D). These findings strongly support the notion that translation of TTP mRNA is regulated by a mechanism similar to that described for TNF mRNA.

## Discussion

**Figure 7:**
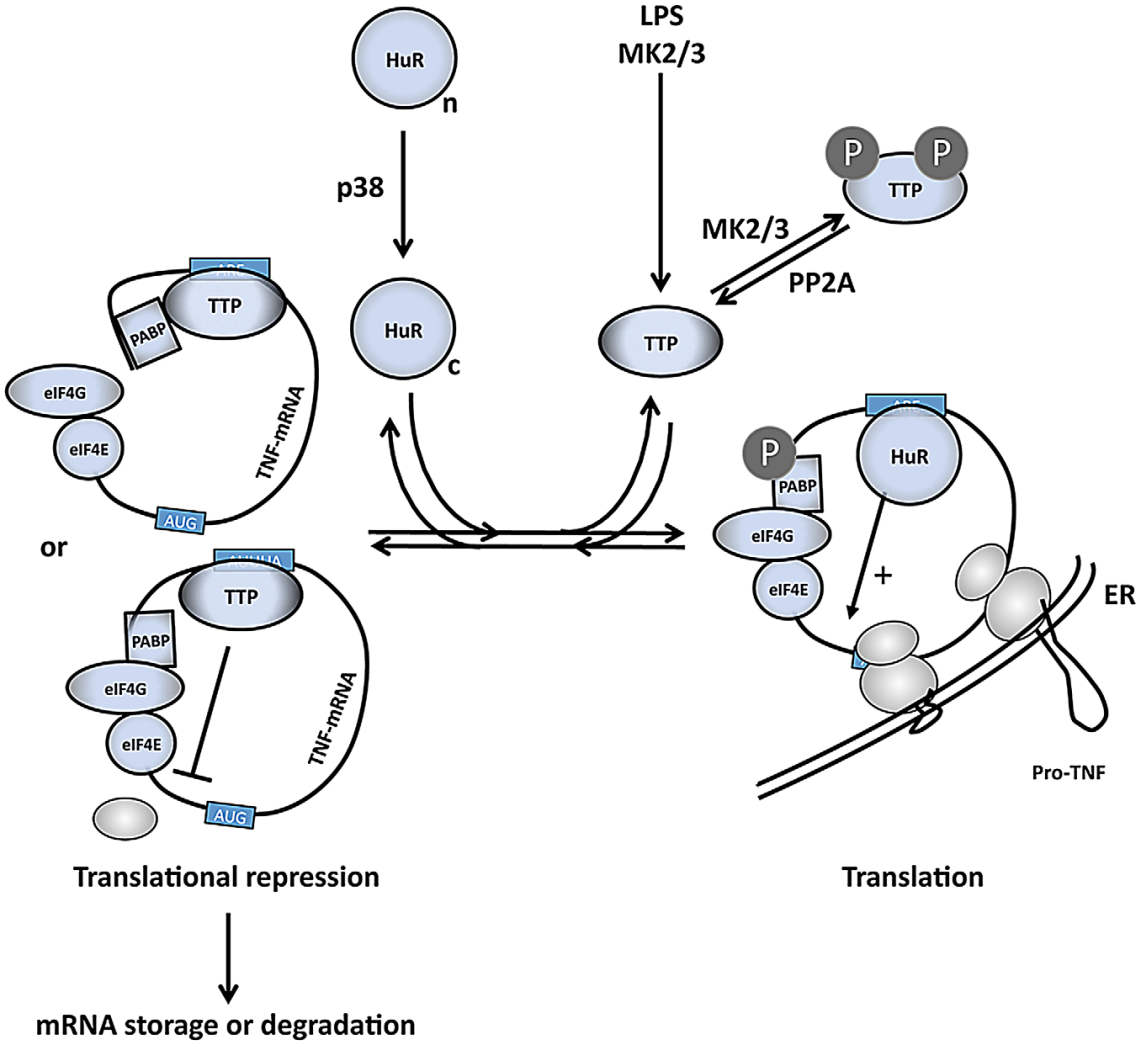
A comprehensive model of the regulation of TNF mRNA and TNF release by p38 and MK2/3. LPS induces expression of TTP and activates p38 and MK2/3. TTP and PABP1 are MK2 substrates [11], [14], [15], while HuR is a direct substrate for both p38 and MK2 and is shifted in its subcellular localization from the nuclear (n) to the cytoplasmic (c) compartment after phosphorylation [47], [74]. HuR binds to the ARE and stimulates initiation of translation. Phosphorylation of TTP by MK2/3 impairs its capacity to compete with cytoplasmic HuR at the ARE in the 3′ UTR of translational controlled mRNAs (right part of scheme). Without phosphorylation at its MK2/3 sites TTP replaces HuR and interferes with translational initiation by competition with eIF4G binding to PABP1 or by inhibition of binding of the 43S PIC. Thereby TTP limits expression of ARE-containing mRNAs, including its own mRNA, at later stages in the response (left part of scheme).

We provide evidence that translational control of TNF-mRNA in macrophages might be achieved by a phosphorylation-regulated competitive mRNA association of the two ABPs TTP and HuR. In this scenario TTP acts as a translational repressor while HuR is a translational activator. By LPS-induced MK2-mediated phosphorylation of TTP the competitive binding equilibrium between TTP and HuR is shifted towards HuR and leads to a stimulation of translation (Figure 7). This mechanism is not restricted to the translation of the type II membrane protein pro-TNF, but is also valid for the translation of the cytosolic protein TTP itself, indicating that this regulation by ABP exchange occurs in an early common step of both processes, probably during translational initiation. This notion is strengthened by the observation that knockdown of HuR leads to an accumulation of cytosolic TNF- and TTP-mRNPs (Figure 4H and Figure 6) and to a reduction of TNF mRNA-containing monosomes (Figure 4H).

In regard to the possible molecular mechanisms of translational initiation (reviewed in [52]), the binding of TTP to the ARE in the 3′ region of the mRNAs probably interferes with eIF4G-dependent mRNA circulation or with the binding of the 43S pre-initiation complex (PIC) to the mRNA (Figure 7). Since no significant accumulation of mRNAs in the 43S-fraction is observed, inhibition of 43S PIC scanning or of joining of the 60S ribosomal subunit seems unlikely. Furthermore, it is not clear whether TTP directly interacts with components of the general translation machinery or whether it recruits further proteins which interfere with translational initiation. From the analysis of TTP's mRNA destabilizing function it is known that TTP can act as a binding platform for various proteins involved in mRNA decay [53]. Furthermore, an mRNA-dependent interaction between TTP and poly(A)-binding proteins (PABPs) has been detected [19], [53], [54] and, interestingly, a direct interaction of TTP and PABP C1 was recently demonstrated in a Y2H screen [55]. Hence, one may speculate that TTP-PABP-interaction could interfere with PABP-eIF4G-interaction and could prevent PABP-eIF4G-eIF4E-mediated circularization of the mRNA as a prerequisite for translation (Figure 7). Taking into account that PABP-1 is also a direct substrate of MK2 [14], its phosphorylation by MK2 could further contribute to the weakening of the TTP-PABP-interaction.

The molecular mechanism of the phosphorylation-driven change in the competitive binding equilibrium of HuR and TTP at the ARE is probably based on the fact that the affinity of TTP is reduced after phosphorylation. However, one should also take into account that cytoplasmic concentrations of HuR and, especially, of the immediate early gene TTP [31] are highly flexible and contribute to the binding equilibrium. A possible scenario of LPS-stimulated regulation could be that newly synthesized TTP is prevented from ARE binding by early phosphorylation via MK2/3 allowing efficient translation of the target mRNAs by the help of HuR. Subsequent decrease in MK2-activity, which peaks after a mere 30 min period, and dephosphorylation of TTP by protein phosphatase 2A [56] could then lead to increased binding of TTP to the ARE, resulting in feedback regulation by translational arrest and destabilization of the target mRNA. Upon LPS-stimulation of macrophages, RNA-IP experiments indicate an early MK2-dependent shift of the binding equilibrium towards HuR supporting the idea that the regulatory mechanism proposed might be relevant *in vivo*.

Recently, a translational repression by TTP has been demonstrated using reporter constructs carrying an ARE in the 3′ UTR in transfected 293T HEK cells [57]. In this system, TTP cooperates with the general translational repressor RCK/P54, which belongs to the DEAD-box helicase family and displays ATP-dependent RNA-unwinding activity [58]. Hence, it cannot be excluded that RCK/p54 also contributes to TTP-dependent translational repression of TNF mRNA in macrophages. The ubiquitin E3 ligase cullin 4B is a recently identified TTP interacting protein which slightly changes polysome loading of TNF mRNA [59]. However, it is also possible that cullin 4B is involved in ubiquitination and subsequent degradation or functional modification of TTP, as already known for its MEKK1-induced TRAF-2-mediated K63 ubiquitinylation [60].

It has also been described that TTP-facilitated binding of miRNAs, such as miR16 or miR369-3, are involved in regulation of the decay of ARE-containing mRNAs [41] and stimulation of translation of TNF mRNA upon serum starvation [42], respectively. A recent screen for miRNAs, which target the 3′ UTR of TNF mRNA, revealed miR-125b and miR-939 as further candidates. However, repression of these miRNAs did not influence TNF mRNA expression, making it unlikely that these miRNAs regulate translation of TNF mRNA [61]. Furthermore, knockdown of Ago2, a target of MK2 and an essential component of the miRNA system, does not influence LPS-induced TNF translation in macrophages (Figure S3). This makes it rather unikely that miRNAs regulate MK2-dependent, LPS-induced translation of TNF mRNA in macrophages.

It is known that TTP destabilizes ARE-containing cytokine mRNAs of IL-1ß, IL-2, IL-3, IL-6, IL-10, TNF, GM-CSF and that, in many cases, this destabilization is relieved by the p38 pathway (reviewed in [16]). Even for the global LPS-stimulated regulation of mRNA decay by TTP it has been convincingly shown that the activity of the p38 pathway inversely correlates with TTP's mRNA degrading activity which arises about 3–9 h after LPS-treatment when p38/MK2/3 activity is back to basal levels [62]. The p38/MK2/3-dependent replacement of TTP by HuR at ARE-containing mRNAs would provide a simple and elegant explanation for this regulation: The binding of TTP to a specific mRNA is the prerequisite for its constitutive degradation. When TTP is displaced from the ARE by HuR (or other ABPs), the specific mRNA is no longer targeted to the constitutive degradation pathway and stabilized. It is interesting to note that the stability of KC mRNA is also regulated by TTP [63], even in a MK2-dependent manner [32] (Figure S8A, S8B), while KC mRNA is not translationally regulated by MK2 (Figure 2D) and we do not observe disappearance of the majority of KC mRNA from the polysome fraction upon HuR knockdown (Figure S8C). KC mRNA contains three isolated AUUUA motifs and two doubles of AUUUAUUUA, which are sufficient for TTP binding and regulation of stability [64]. Interestingly, the isolated AUUUA motifs of KC mRNA are not sufficient for HuR binding. Furthermore, although at least its AUUUAUUUA stretch fits rather well to the HuR consensus UUUUUUU, there is no HuR binding reported for KC mRNA in the transcriptome wide screens [65], [66]. Hence, KC is an example of separation of TTP function from HuR action, since HuR is not able to compete with TTP binding to KC mRNA and is not necessary for its translation.

TTP has been postulated as the essential component of the feedback regulation of the LPS-stimulated TNF-response [29]. Increased TTP phosphorylation by MK2, which neutralizes TTP repressor function [15], [30], is paralleled by transcriptional activation of the TTP gene, which belongs to the group of immediate early genes (Figure 1A and [67]), as a result of phosphorylation of the transcription factor SRF by MK2 [31]. Interestingly, the feedback regulation by TTP also comprises TTP's binding to an ARE in the 3′UTR of its own mRNA [51]. Here, we have demonstrated that translation of TTP mRNA is also stimulated by MK2, probably by the same mechanism of ARE-replacement of phospho-TTP by HuR at the ARE of TTP mRNA, since knockdown of HuR inhibits translation and protein expression of TTP (Figure 6). Hence, MK2 not only stimulates transcription but also translation of TTP and rapidly enables the re-synthesis of non-phospho-TTP, which again limits TNF expression and TTP expression itself. The parallel translational stimulation of TNF and TTP by the same MK2-dependent mechanism for the first time explains the paradox observation that both, TNF biosynthesis and TTP expression are strongly reduced in MK2-deficient macrophages [32], whereas complete deletion of TTP leads to increased TNF biosynthesis [29]. In addition, since both TNF and TTP are further reduced in MK2/3 double-deficient macrophages when compared to MK2-deficient cells, it is highly likely that MK3 shares these functions of MK2.

The fact that ABPs interact with their cognate mRNAs and regulate their own expression has been comprehensively described [68]. Furthermore, a complex network of post-transcriptional cross-regulation of expression between ABPs such as HuR, TIA-1, KSRP, AUF1 is known to exist [68]. However, in the experimental system of immortalized macrophages applied in this study, we could not detect significant changes in expression of other ABPs (TIA-1, KSRP) as a result of knockdown of TTP and HuR. Furthermore, the binary *in vitro*-binding system using purified HuR and TTP proteins, which is sufficient to observe the MK2-dependent exchange between TTP by HuR, suggests that the regulatory mechanism postulated could function without the involvement of other ABPs apart from TTP and HuR. On the other hand, specific binding of isoforms of the mRNA-binding protein AUF1 to TTP has been described [69]. Interestingly, this interaction increases RNA-binding affinity of TTP in an *in vitro* assay about five-fold. Hence, it cannot be excluded that further ABPs modulate the proposed regulation *in vivo*.

HuR is predominantly held responsible for constitutive stabilization of ARE-containing mRNAs [20] and demonstrably binds to the ARE of TNF mRNA [22], [23]. Besides the TNF mRNA stabilizing function, HuR has also been described to influence translation. In macrophages, HuR acts as a homeostatic coordinator of expression of ARE-containing mRNAs [26], which, when deleted, also increases inflammatory cytokine production of macrophages. This effect could be explained by the fact that HuR is essential for efficient expression of TTP (Figure 6) necessary for down-regulation of the inflammatory response. On the other hand, LPS-stimulated TNF production is significantly inhibited in the NZW mouse strain containing two different 3-base insertions in the TNF-ARE, which inhibit binding of HuR to the ARE [70]. This finding indicates a positive role of the specific binding of HuR to the TNF-ARE for TNF expression and is in agreement with our observations that expression of HuR is necessary for efficient translation of TNF. It is interesting to note that stress-induced release of miR-122-mediated translational repression of CAT-1 mRNA also requires HuR [71] indicating a more general role of HuR in counteracting translational repression, not only by ABPs but also by miRNAs.

HuR is mainly localized in the nucleus but continuously shuttles between cytoplasm and nucleus [72]. There are various post-translational modifications of HuR described, which result in changes in its subcellular distribution [73]. Interestingly, p38 phosphorylates HuR at T118 in a stress-dependent manner resulting in significant cytoplasmic accumulation of HuR [47]. Expression of constitutively active MK2 also leads to cytoplasmic accumulation of endogenous HuR [74]. This p38/MK2/3-stimulated increase in the cytosolic HuR concentration could be a prerequisite for translational stimulation of ARE-containing mRNAs by HuR (cf. Figure 7). Hence, the p38/MK2 pathway may regulate specific translational initiation by catalytic activity of both, p38 and MK2, in parallel at the levels of translocation and regulation of activity of ABPs.

## Experimental Procedures

### Immortalization and Retroviral Rescue of MK2/3-Deficient Macrophages

Primary MK2/3 DKO BMDMs were immortalized as previously described [75]. Retroviral transduction of MK2/3 immortalized BMDMs with vectors encoding MK2 wild type, kinase dead MK2 (K76R) and the empty vector (GFP) was carried out as described [31], [75].

### Cell Culture and Treatments

Immortalized and retroviral transduced MK2/3 DKO BMDMs were grown in DMEM containing 10% FCS, 2 mM L-glutamine, 100 U penicillin G/ml, 100 mg streptomycin/ml and 0,1 mM non essential amino acids mixture (Life Technologies/Invitrogen) under humidified conditions with 5% CO_2_ at 37°C. HEK293T cells were grown under the same conditions and were transfected with the calciumphosphate method. The p38 inhibitor SB202190 (Sigma) and LPS (*Escherichia coli* 0127:B8, Sigma) were used at concentrations of 5 µM and 1 µg/ml, respectively. Primary BMDM were derived as previously described by using 10 ng/ml M-CSF (Wyeth/Pfizer) [30].

### siRNA Knockdown

siRNA-mediated knockdown in BMDMs was performed following the instructions of the protocol for knockdown in RAW264.7 cells using HiPerFect transfection reagent (Qiagen). 8×10^4^ cells were seeded in 100 µl growth medium in a 24-well plate. Appoximately 187.5 ng siRNA was mixed with 3 µl HiPerFect and 100 µl Opti-MEM (Life Technologies/Invitrogen) and incubated for 5 minutes at room temperature. The mixture was added dropwise to the wells and after 6 hrs of incubation 400 µl complete growth medium was added. The next day the medium was changed to complete medium. The highest efficiency for the different knockdown experiments was achieved after 48 hrs of siRNA treatment. For siRNA transfections of 10 cm cell culture plates the protocol was upscaled according to the instructions of the HiPerFect handbook (Qiagen). The following mouse specific siRNAs (target sequences) were used (Qiagen): siTTP: 5′-CCTGAGAATCCTGGTGCTCAA-3′ (Mm_Zfp36_6), siHuR: 5′-CAGAAACATTTGAGCATTGTA-3′ (Mm_Elavl1_4), siAgo2: 5′-CACTATGAATTGGACATCAAA-3′. For control knockdown Allstars negative Control siRNA (Qiagen 1027281) was used.

### Western Blotting and ELISA

Western blotting was performed as described [31]. Blots were developed with an ECL detection kit (Santa Cruz Biotechnology) and digital chemoluminescence images were taken by a Luminescent Image Analyzer LAS-3000 (Fujifilm). Primary antibodies used were: anti-eEF2 (2332), anti-Histone3 (9715), anti-MK2 (3042), anti-pMK2pT222 (9A7) (3316), anti-p38 (9212), anti-pp38pT180/pY182 (9211), anti-pPKD (4381B) and anti-S6 (5G10) 2217 (all from Cell Signaling), anti-Ago2 (M01) (Abnova), anti-GAPDH (6C5) Mab374 (Millipore/Chemicon), anti-ß-Actin (C4) sc-47778, anti-GFP (B-2) sc-9996, anti-HuR (19F12) sc-56709, anti-Msk1 (H-19) sc-9392, anti-TIA-1 (C-20) sc-1751, anti-TNF (L19) sc-1351 (all from Santa Cruz Biotechnologies). Antibodies against KSRP [76], TTP (SAK21B) [77], TTP-pS178 [56] and NOGO-B [35] were described previously and were kindly provided by Drs. A. R. Clark (London), G. Stoecklin (Heidelberg) and P. Cohen (Dundee), respectively. Streptactin-HRP conjugate (IBA BioTAGnology) and secondary HRP-conjugated antibodies (Santa Cruz Biotechnologies) were used. The mouse TNF-alpha Ready-SET-Go! kit from eBiosciences (88–7324) was used for TNF ELISA. Quantification of TNF and ß-actin Western blot signals was performed with the Multi Gauge 3.2 software (Fujifilm). For each individual experiment two different exposure times were analyzed and then normalized to the ß-actin signals. Two independent Western blot experiments with different sample loading were carried out. For inter-experimental comparison the signals were normalized by the intensity of non-stimulated MK2-rescued cells transfected with control siRNA.

### Saponin Fractionation

BMDMs of a 6 cm plate of 80% confluence were dissolved in 150 µl extraction buffer (20 mM Tris pH 8.0, 140 mM KCl, 5 mM MgCl2, 0.5 mM DTT, 0.1 mg/ml cycloheximide and 0.5 mg/ml Heparin). Then 1% (w/v) Saponin (ICN chemicals) dissolved in DMSO was added to a final concentration of 0.1% (v/v) followed by 20 minutes incubation on ice with partial vortexing. Cells were then centrifuged 5 minutes at 500×g. The supernatant (cytsosol) was kept separately on ice. The pellet (microsomes and nuclei) was washed with extraction buffer and was centrifuged again (500×g). The pellet was dissolved in extraction buffer containing 0.5% v/v NP-40 (Fluka). Both cytosol and dissolved microsomal fraction were then centrifuged 10 minutes at 7500×g. The resulting supernatants represent the cytosolic and the microsomal ER fraction free of nuclear components.

### Polysome Profiling by Density Gradient Centrifugation and qRT–PCR

1×10^7^ BMDMs were used for a single gradient experiment. The cells were washed twice with 1×PBS containing 0.1 mg/ml cycloheximide and differentially lysed as described above. For RNaseA treatment controls, 1.5 mg/ml RNaseA (Carl Roth) was added to the lysates for 15 min on ice. The cytosolic and ER ribosome extracts (400 µl each) were loaded on linear sucrose gradients (12 ml) ranging from 50% (bottom) to 10% (top) sucrose containing 140 mM KCl, 20 mM Tris pH 8, 5 mM MgCl_2_, 0.1 mg/ml cycloheximide, 0.5 mg/ml Heparin and 0.5 mM dithiothreitol (DTT). After loading, the extracts were separated by ultracentrifugation in a SW40.1 Ti Rotor (Beckmann-Coulter) for 2 hrs at 35000 rpm and 4°C. Subsequently, 12 gradient fractions (each 1 ml) were collected using a UA-6 UV/VIS device (Teledyne/ISCO Inc.) that was connected to an optical unit allowing OD documentation. RNA isolation from the gradient fractions was performed by adding 1/10 volume of 3 M Na-acetate (Sigma) and 1 volume of isopropanol and overnight precipitation at −20°C. RNA was pelleted by centrifugation at 13000×g for 20 min at 4°C. For the analysis of co-sedimenting proteins, trichloro acetic acid (TCA) was added to each fraction (final 10% v/v). Proteins were precipitated overnight at 4°C and subsequently centrifuged for 20 min at 13000×g at 4°C. Pellets were washed twice with ice cold acetone, dissolved in 100 µl 2×SDS-loading buffer and heated for 5 minutes at 95°C.

RNAs were isolated by resolving the cells or pellets obtained from polysome gradient fractions in lysis buffer RA1 of the RNA NucleoSpin II kit and subsequent processing (Macherey+Nagel). For cDNA synthesis, the cDNA first strand cDNA synthesis kit (Thermo/Fermentas) was used. cDNAs were diluted 1∶20 for detection in qRT-PCR reactions. For detection of TTP and KC cDNA predesigned and FAM-labelled TaqMan primer mixtures from Applied Biosystems were used (Mm00457144_m1 Zfp36, Mm00433859_m1 Cxcl1). TNF cDNA was amplified using a labelled probe (5′-FAM – CAC GTC GTA GCA AAC CAC CAA GTG GA – BHQ1-3′) together with flanking primers (forward: 5′-CAT CTT CTC AAA ATT CGA GTG ACA A-3′ and reverse: 5′-TGG GAG TAG ACA AGG TAC AAC CC-3′). ß-actin cDNA was amplified with the VIC-labelled predesigned probe from Applied Biosystems (4352341E) allowing two channel detection of one cDNA. Amplifications were carried out in a 1× SensiFAST Probe No-ROX buffer system (Bioline) using a Rotor-Gene-Q device (Qiagen). The threshold cycle (CT) of each individual PCR product was calculated by the software of the instrument.

### Purification of Strep-Tagged Proteins

15 cm diameter plates of HEK293T cells were transfected with the expression constructs (pcDNA3-His-Strep-TTP, -TTP-S52A, -TTP-S178A, -TTP-AA (S52,178A) and pEXPR-IBA105-HuR) and lysed 24 hrs post transfection. Strep-tagged HuR and TTP protein was purified by affinity chromatography using streptactin beads (IBA TAGnologies) as described previously [76]. Proteins were eluted with desthiobiotin (IBA TAGnologies) and the protein concentration was determined by Coomassie staining of the bands in SDS-PAGE compared to BSA standards using the Multi Gauge quantification software 3.2 (Fujifilm). Dephosphorylation of purified proteins was achieved by incubation with calf intestinal phosphatase (CIP) for 15 minutes at 37°C.

### RNA–EMSA

Strep-tag purified proteins were dissolved in EMSA-shift buffer (20 mM HEPES pH 7.6, 3 mM MgCl2, 40 mM KCl, 5% Glycerol, 2 mM DTT, 4 µg tRNA) to give a total volume of 20 µl and were incubated with 75 fmol of an 5′-DY681-labbeled AU-rich RNA-probe (5′-AUU UAU UUA UUU AUU UAU UUA UUU A-3′) for 25 minutes at 4°C. For supershift experiments 0.2 µg of specific antibody was added to the mixture after 10 min of preincubation at 4°C. The reaction mix was then loaded onto a 4% native shift gel after a pre-run of 30 minutes at 80 V and 4°C and separated at 80 V for 90 minutes in 0.25× TBE buffer at 4°C. The detection of RNA-protein complexes was performed by visualization of DY681 on a LiCOR Odyssey infrared-scanner.

### Competitive ARE-Binding Assay

As competition assay, purified HuR protein (25–300 ng) was first incubated together with 75 fmol of the 5′-DY681-labelled AU-rich RNA-probe (see above) in a 20 µl reaction mix in 96-well plates. Where indicated, 300 ng of the purified recombinant protein kinases His_6_-MK2 (in 1.0 µl) and GST-p38 (in 0.3 µl) (Menon et al. 2010) and 0.5 µl 10 mM ATP were added. After 10 min at 30°C, purified TTP protein was added and incubated for another 15 min minutes at 30°C. Then, the RNA-protein crosslink was performed by UV auto cross-linking using a Stratalinker (Stratagene). The cross-linking products were separated by SDS-PAGE and detected using the LiCOR Odyssey infrared scanner.

### *In Vitro* Kinase Assay

For detection of phosphorylation, strep-tag purified proteins were mixed together with *E.coli*-expressed and purified His_6_-MK2, GST-p38, GST protein and radiolabelled gamma-^33^P-ATP as described previously (Menon et al. 2010). Purified recombinant Hsp25 served as a positive control for MK2 kinase activity. Samples were resolved by SDS-PAGE and analyzed by phospho-imaging on a FLA-5000 (Fujifilm) system.

### RNA–IP

MK2-rescued and GFP-transduced macrophage lines were stimulated with LPS for 1 h, UV treated for 30 seconds (120 mJ/m^2^) and subsequently lysed in buffer containing 30 mM HEPES (pH 7.4), 150 mM NaCl and 0.5% v/v NP-40 with protease inhibitors. For RNA-IP, 1 µg of anti-HuR (mouse IgG_1_) and, as negative control, 0.5 µg of anti-GFP (mouse IgG_2a_) were incubated overnight at 4°C with the same amounts of cross-linked lysates in a volume of 0.5 ml. Afterwards 30 µl of Protein G Sepharose (GE Healthcare) suspension blocked with 50 µg/ml t-RNA for 1 h were added. After further incubating 2 h at 4°C, the beads were washed extensively in lysis buffer containing 0.25% v/v NP-40. The associating RNAs were eluted by vigorous vortexing of the beads in 350 µl RA1 lysis buffer (RNA NucleoSpin II kit (Macherey+Nagel)). Samples of the total lysates (input) and the re-dissolved precipitates were then analyzed by qRT-PCR.

## Supporting Information

Figure S1 Comparison of MK2 expression in the rescued cell line, wild type BMDMs and RAW264.7 cells by Western blot analysis. Lysates of the MK2/3-deficent cell lines rescued with MK2 (DKO+MK2) or transduced with GFP (DKO+GFP; negative control), of immortalized wild type BMDM and of the macrophage cell line RAW 264.7 were applied to Western blotting using anti-MK2 antibodies. Loaded samples correspond to 20 µg of total lysate proteins. Equal loading was monitored by immunostaining of eEF2 and GAPDH. The slower migrating band of endogenous MK2 probably corresponds to a differentially spliced isoform of MK2.
(PDF)

Figure S2 Catalytic activity of MK2 is necessary for the translational regulation of TNF in reconstituted macrophage cell lines. MK2/3-deficient macrophages were immortalized as described and the immortalized cell line was transduced with retroviral constructs coding for MK2 and GFP (+MK2) or catalytic-dead MK2-K79R and GFP (+MK2-K79R), respectively. A) Western blot analysis of LPS (1 µg/ml)-induced protein expression and phosphorylation of the protein kinases p38 and MK2. MK2 and the mutant MK2-K79R are expressed to comparable levels. In contrast to MK2-rescued “wild type” cells, cells rescued by the catalytic-dead mutant cells display absence of NOGO-B phosphorylation and a reduced TTP concentration. B) Relative TNF mRNA level in MK2- and MK2-K79R-rescued as well as in GFP only-transduced cells 1 h after LPS treatment as determined by RT-qPCR of TNF and ß-actin mRNA does not differ significantly. C) Comparison of the amount of released TNF protein between MK2- and MK2-K79R-rescued and GFP-transduced cells by ELISA 4 h after LPS stimulation. P values of the two-tailed student's t-test are given.
(PDF)

Figure S3 MK2 does not affect polyadenylation of TNF mRNA in LPS-stimulated macrophages. Poly(A) tail length of TNF mRNA was determined in MK2/3 double-deficient macrophages rescued with MK2 (+MK2) or transduced with GFP (+GFP) 1 h after LPS-stimulation using the USB poly(A) tail-length assay kit (Affymetrix) and a TNF-specific forward primer (5′-TCTTAATAACGCTGATTTGGT-3′) generating a fragment of 111 bases+poly(A) tail (Poly(A)+RT). As positive control, a 150 bp fragment of the TNF 3′UTR was amplified (TNF+RT). As negative controls, the reactions were carried out without prior reverse transcription (−RT). The poly(A) tail length is estimated to be between 60–200 bases in both cell lines.
(PDF)

Figure S4 MK2-dependent TNF mRNA distribution between cytosolic and ER fraction. Copy numbers of TNF mRNA (A) and ß-actin mRNA (B) were determined by qRT-PCR of cytosolic and ER fractions of lysate from equal amounts of MK2-rescued (+MK2) or GFP-transfected (+GFP) cells stimulated by LPS treatment for 1 h. p values of the two-tailed student's t-test are given.
(PDF)

Figure S5 Selected polysome profile analyses and their biological repeats. Two independent experiments (Experiment 1, Experiment 2) of cell treatment, subsequent polysome profiling including analysis of TNF mRNA distribution are shown for the key experiments of Figure 2B and Figure 4C, 4G, 4K. In addition, the polysomal (fractions 6–11)/non-polysomal (fractions 2–5) TNF-mRNA ratios (ratio P/M) are shown on the right side of each profile.
(PDF)

Figure S6 Ago2 does not affect LPS-induced MK2-dependent translation of TNF mRNA in macrophages. Knockdown of Ago2 in MK2-rescued and GFP-transduced macrophages, corresponding Western blot analysis of knockdown efficiency on expression of Ago-2 in macrophages (−siAgo2 = siCon−scrambled siRNA, +siAgo2−Ago2 specific siRNA) and polysome profiles for TNF mRNA of the ER fraction of LPS-stimulated GFP-transduced and MK2-rescued cells treated with siAgo2 or scrambled control (+siCon).
(PDF)

Figure S7 Control experiments for the in vitro ARE-binding assay. A) Different amounts (in ng) of strep-tagged HuR and TTP expressed in and purified from HEK293T cells analyzed by Western blot using strep-tactin-labeled horseradish peroxidase (strep-tactin-HRP). B) Catalytic activity of MK2 is necessary and catalytic activity of p38 is not sufficient for the TTP-HuR-exchange. C–E) Expression and analysis of the TTP mutants where the phosphorylation sites for MK2, serine 52 and 178, are replaced by alanine. C) TTP phosphorylation site mutants expressed in and purified from HEK293T cells analyzed by Western blot using strep-tactin-labeled horseradish peroxidase (strep-tactin-HRP) and an phospho-S178-TTP antibody. Cells were left untreated or stimulated by for 0.5 h by 10 µg/ml anisomycin to activate p38 MAPK and MK2/3. As a negative control, p38 stimulation was performed in the presence of 5 µM SB202190 – a specific inhibitor of p38. D) Analysis of ARE-binding of the TTP-AA mutants in the absence (+GST+GST-p38) and presence of catalytic activity of MK2 (+GST-MK2+GST-p38) by EMSA. E) Competitive binding of HuR (25 and 50 ng) and TTP and its mutants (100 ng) in the absence (GST-MK2) and presence of catalytic activity of MK2 (GST-p38, GST-MK2). Only wild type TTP can be efficiently replaced by HuR under these experimental conditions. B, E) Infrared images of proteins cross-linked to the ARE probe and separated in SDS-Page.
(PDF)

Figure S8 MK2-dependent stability and HuR-independent translation of KC mRNA. A) KC mRNA decay after 1 h of LPS treatment and subsequent inhibition of transcription using 5 µg/ml actinomycin D in MK2-rescued (+MK2) and GFP-transduced MK2/3-deficient immortalized macrophages (+GFP). mRNA levels immediately before addition of Act D were set to 100%. mRNA levels were determined by qRT-PCR before and at 30 and 60 min after Act D. Exponential regression analysis is shown. B) KC mRNA levels relative to actin mRNA in MK2-rescued (+MK2) and GFP-transduced MK2/3-deficient cells (+GFP) determined 1 h and 2 h after LPS stimulation. P values of the two-tailed student's t-test are given. C) Polysome profiling for KC mRNA in MK2-rescued cells upon HuR knockdown corresponding to Figure 4G and Figure S5.
(PDF)
