# Supplementary figures and images for: The p38/MK2-Driven Exchange between Tristetraprolin and HuR Regulates AU–Rich Element–Dependent Translation
Source: PLoS Genet. 2012 Sep 27;8(9):e1002977. doi: 10.1371/journal.pgen.1002977 (PMC3459988; doi:10.1371/journal.pgen.1002977)

Figure S1

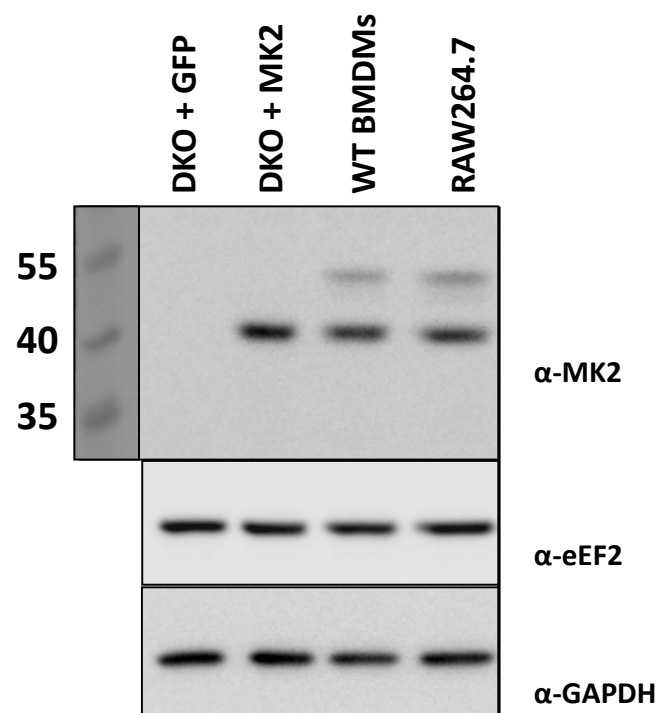

Supplement: Figure S1 — Comparison of MK2 expression in the rescued cell line, wild type BMDMs and RAW264.7 cells by Western blot analysis. Lysates of the MK2/3-deficent cell lines rescued with MK2 (DKO+MK2) or transduced with GFP (DKO+GFP; negative control), of immortalized wild type BMDM and of the macrophage cell line RAW 264.7 were applied to Western blotting using anti-MK2 antibodies. Loaded samples correspond to 20 µg of total lysate proteins. Equal loading was monitored by immunostaining of eEF2 and GAPDH. The slower migrating band of endogenous MK2 probably corresponds to a differentially spliced isoform of MK2. (PDF) [file pgen.1002977.s001.pdf]

Figure S2

A

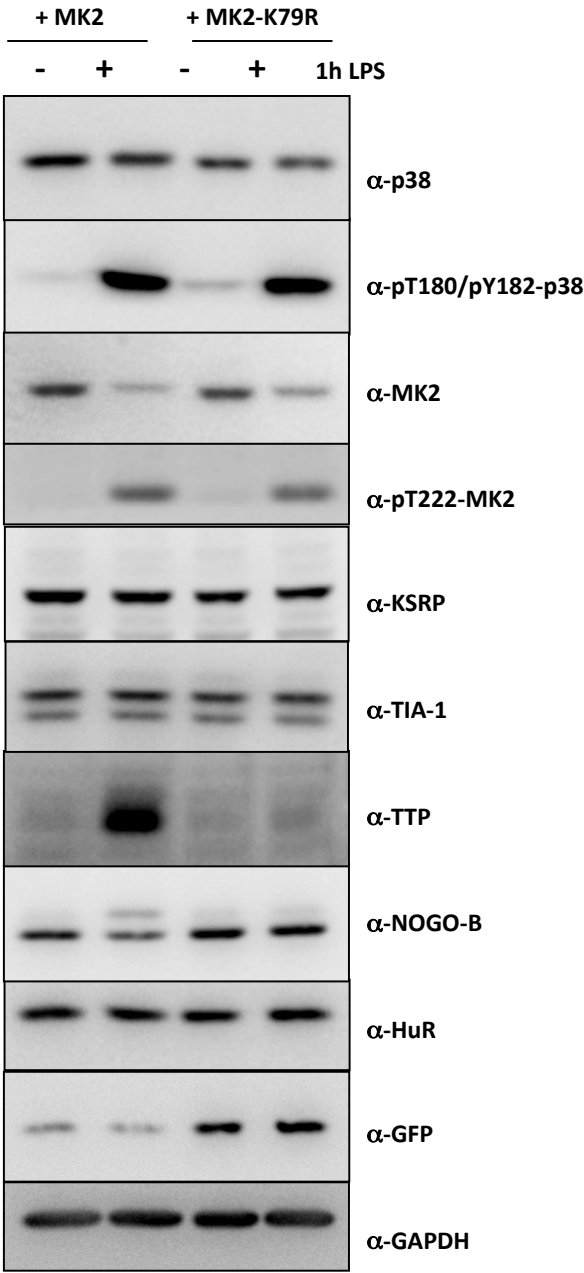

B

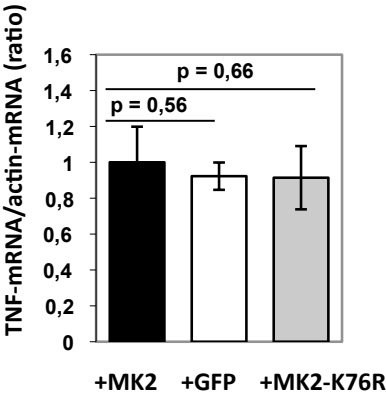

C

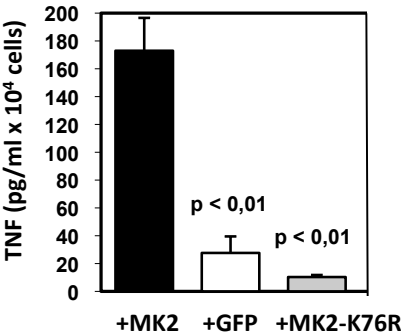

Supplement: Figure S2 — Catalytic activity of MK2 is necessary for the translational regulation of TNF in reconstituted macrophage cell lines. MK2/3-deficient macrophages were immortalized as described and the immortalized cell line was transduced with retroviral constructs coding for MK2 and GFP (+MK2) or catalytic-dead MK2-K79R and GFP (+MK2-K79R), respectively. A) Western blot analysis of LPS (1 µg/ml)-induced protein expression and phosphorylation of the protein kinases p38 and MK2. MK2 and the mutant MK2-K79R are expressed to comparable levels. In contrast to MK2-rescued “wild type” cells, cells rescued by the catalytic-dead mutant cells display absence of NOGO-B phosphorylation and a reduced TTP concentration. B) Relative TNF mRNA level in MK2- and MK2-K79R-rescued as well as in GFP only-transduced cells 1 h after LPS treatment as determined by RT-qPCR of TNF and ß-actin mRNA does not differ significantly. C) Comparison of the amount of released TNF protein between MK2- and MK2-K79R-rescued and GFP-transduced cells by ELISA 4 h after LPS stimulation. P values of the two-tailed student's t-test are given. (PDF) [file pgen.1002977.s002.pdf]

Figure S3

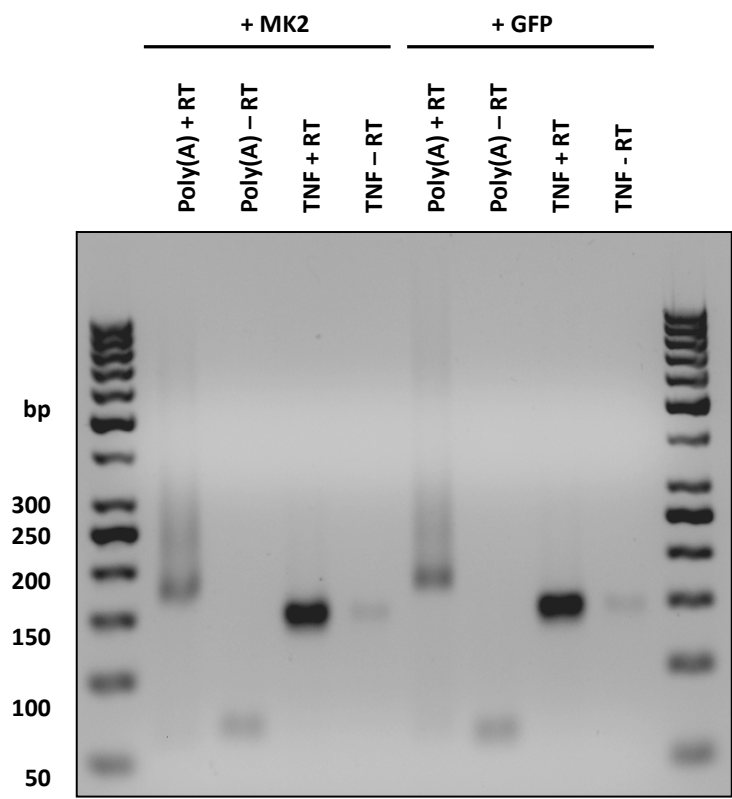

Supplement: Figure S3 — MK2 does not affect polyadenylation of TNF mRNA in LPS-stimulated macrophages. Poly(A) tail length of TNF mRNA was determined in MK2/3 double-deficient macrophages rescued with MK2 (+MK2) or transduced with GFP (+GFP) 1 h after LPS-stimulation using the USB poly(A) tail-length assay kit (Affymetrix) and a TNF-specific forward primer (5′-TCTTAATAACGCTGATTTGGT-3′) generating a fragment of 111 bases+poly(A) tail (Poly(A)+RT). As positive control, a 150 bp fragment of the TNF 3′UTR was amplified (TNF+RT). As negative controls, the reactions were carried out without prior reverse transcription (−RT). The poly(A) tail length is estimated to be between 60–200 bases in both cell lines. (PDF) [file pgen.1002977.s003.pdf]

Figure S4

A

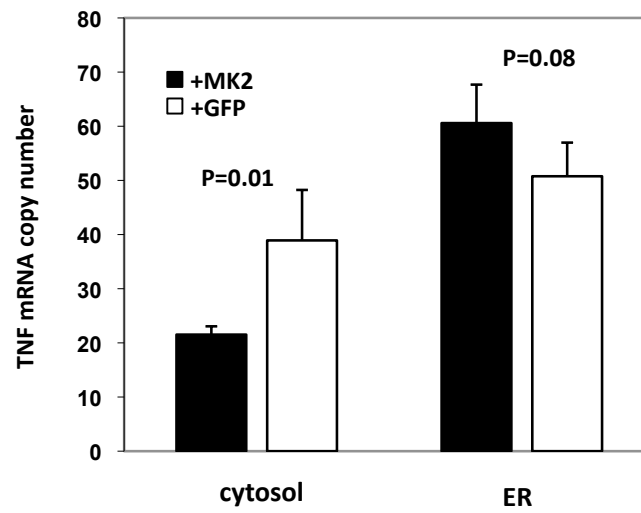

B

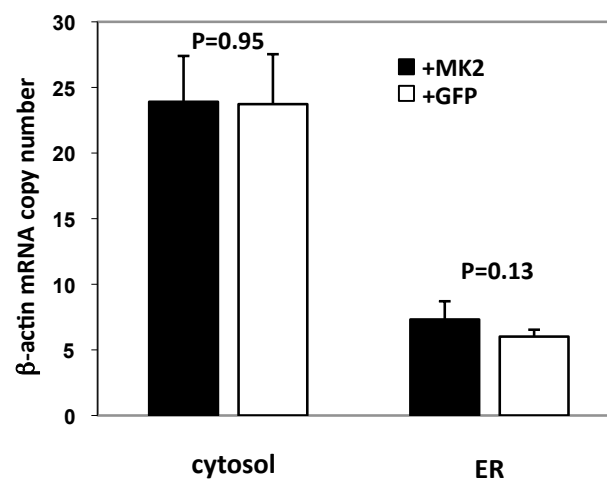

Supplement: Figure S4 — MK2-dependent TNF mRNA distribution between cytosolic and ER fraction. Copy numbers of TNF mRNA (A) and ß-actin mRNA (B) were determined by qRT-PCR of cytosolic and ER fractions of lysate from equal amounts of MK2-rescued (+MK2) or GFP-transfected (+GFP) cells stimulated by LPS treatment for 1 h. p values of the two-tailed student's t-test are given. (PDF) [file pgen.1002977.s004.pdf]

### Experiment 1

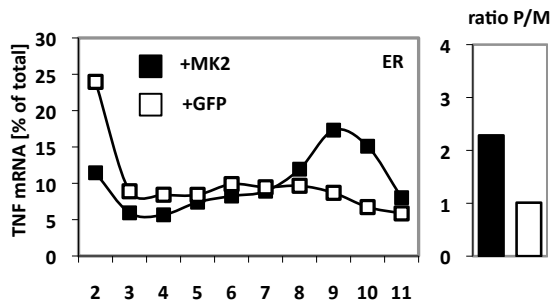

### Experiment 2

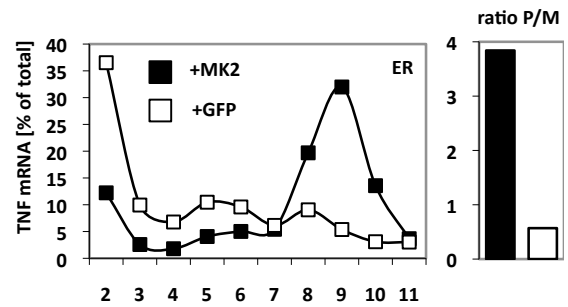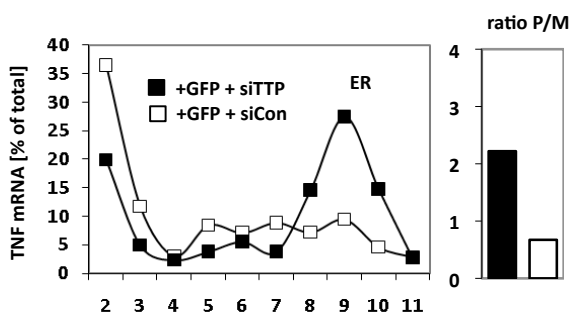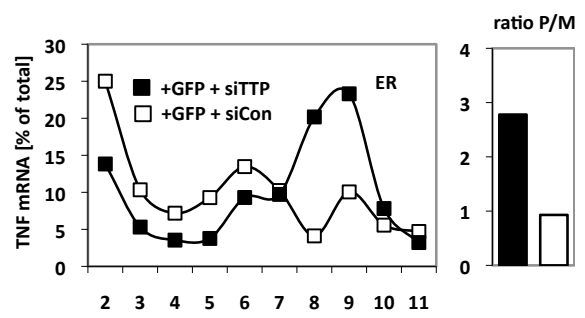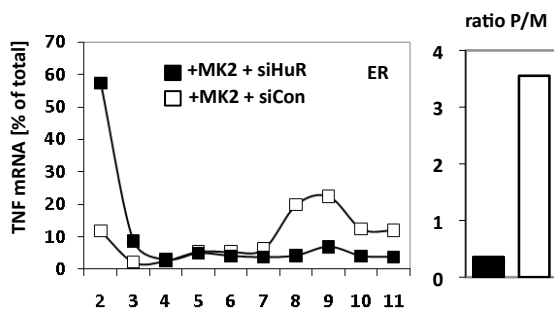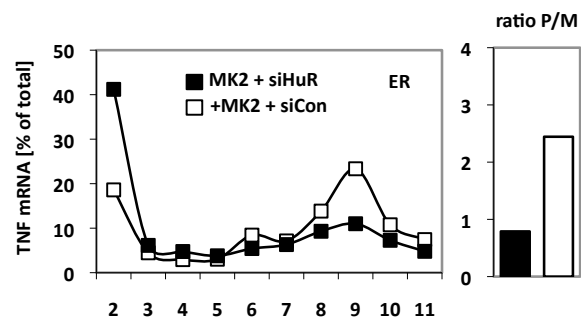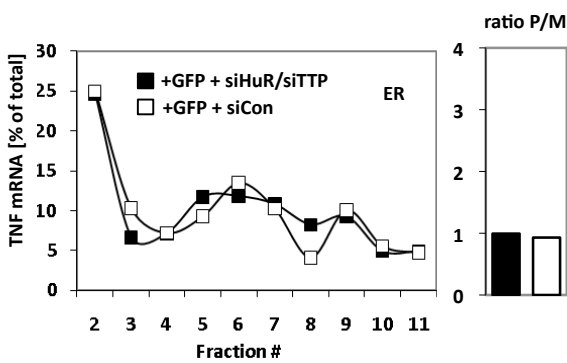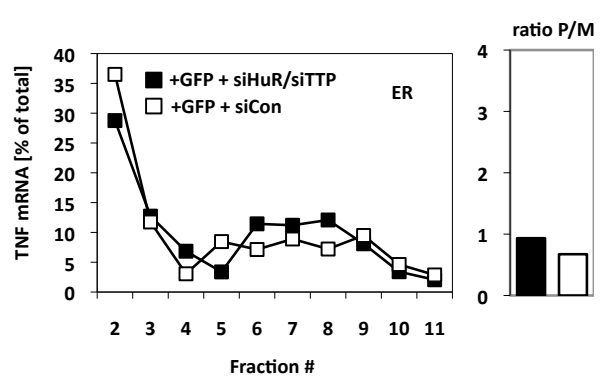

Supplement: Figure S5 — Selected polysome profile analyses and their biological repeats. Two independent experiments (Experiment 1, Experiment 2) of cell treatment, subsequent polysome profiling including analysis of TNF mRNA distribution are shown for the key experiments of Figure 2B and Figure 4C, 4G, 4K. In addition, the polysomal (fractions 6–11)/non-polysomal (fractions 2–5) TNF-mRNA ratios (ratio P/M) are shown on the right side of each profile. (PDF) [file pgen.1002977.s005.pdf]

Figure S6

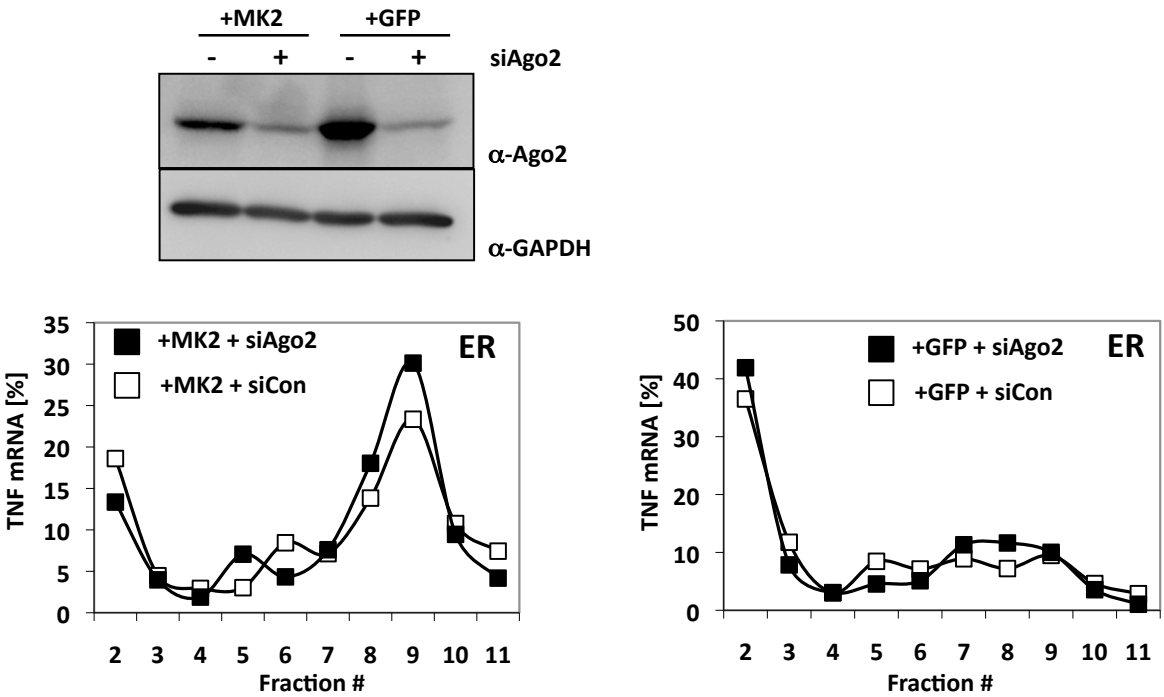

Supplement: Figure S6 — Ago2 does not affect LPS-induced MK2-dependent translation of TNF mRNA in macrophages. Knockdown of Ago2 in MK2-rescued and GFP-transduced macrophages, corresponding Western blot analysis of knockdown efficiency on expression of Ago-2 in macrophages (−siAgo2 = siCon−scrambled siRNA, +siAgo2−Ago2 specific siRNA) and polysome profiles for TNF mRNA of the ER fraction of LPS-stimulated GFP-transduced and MK2-rescued cells treated with siAgo2 or scrambled control (+siCon). (PDF) [file pgen.1002977.s006.pdf]

Figure S7

**A**

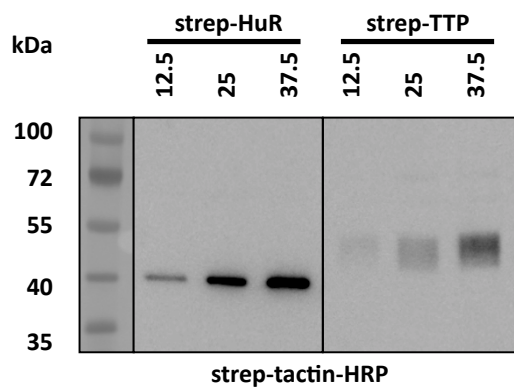

**B**

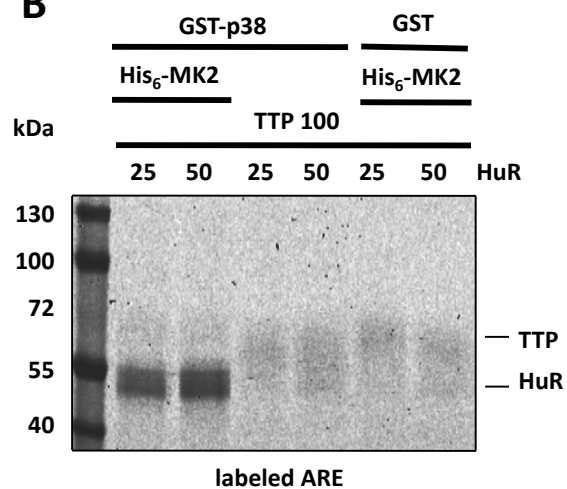

**C**

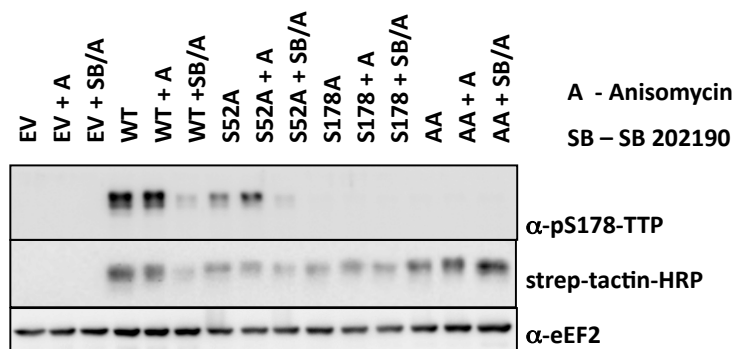

**D**

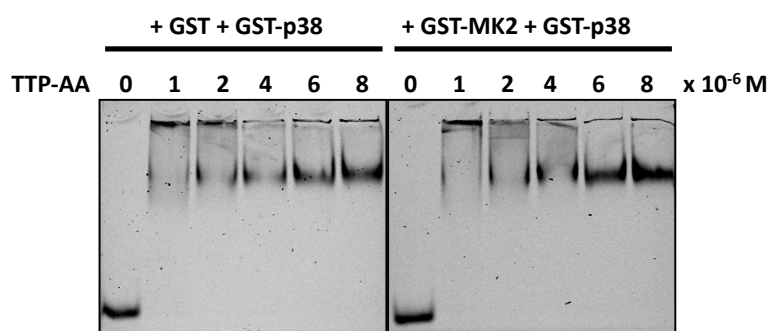

**E**

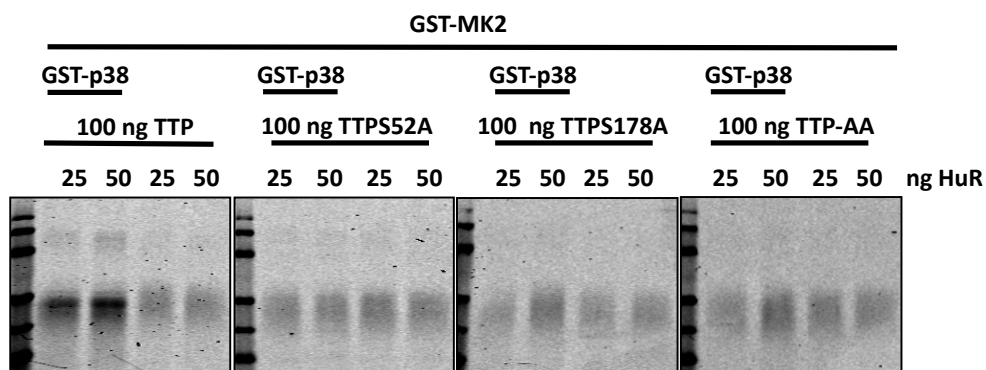

Supplement: Figure S7 — Control experiments for the in vitro ARE-binding assay. A) Different amounts (in ng) of strep-tagged HuR and TTP expressed in and purified from HEK293T cells analyzed by Western blot using strep-tactin-labeled horseradish peroxidase (strep-tactin-HRP). B) Catalytic activity of MK2 is necessary and catalytic activity of p38 is not sufficient for the TTP-HuR-exchange. C–E) Expression and analysis of the TTP mutants where the phosphorylation sites for MK2, serine 52 and 178, are replaced by alanine. C) TTP phosphorylation site mutants expressed in and purified from HEK293T cells analyzed by Western blot using strep-tactin-labeled horseradish peroxidase (strep-tactin-HRP) and an phospho-S178-TTP antibody. Cells were left untreated or stimulated by for 0.5 h by 10 µg/ml anisomycin to activate p38 MAPK and MK2/3. As a negative control, p38 stimulation was performed in the presence of 5 µM SB202190 – a specific inhibitor of p38. D) Analysis of ARE-binding of the TTP-AA mutants in the absence (+GST+GST-p38) and presence of catalytic activity of MK2 (+GST-MK2+GST-p38) by EMSA. E) Competitive binding of HuR (25 and 50 ng) and TTP and its mutants (100 ng) in the absence (GST-MK2) and presence of catalytic activity of MK2 (GST-p38, GST-MK2). Only wild type TTP can be efficiently replaced by HuR under these experimental conditions. B, E) Infrared images of proteins cross-linked to the ARE probe and separated in SDS-Page. (PDF) [file pgen.1002977.s007.pdf]

Figure S8

A

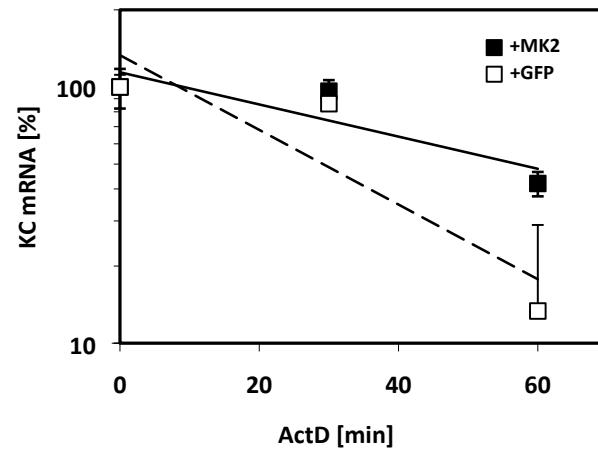

B

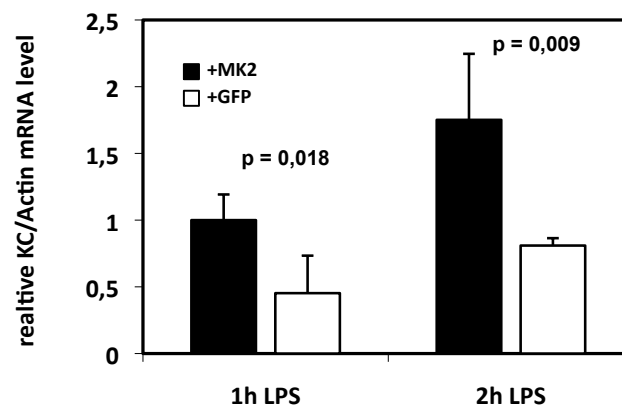

C

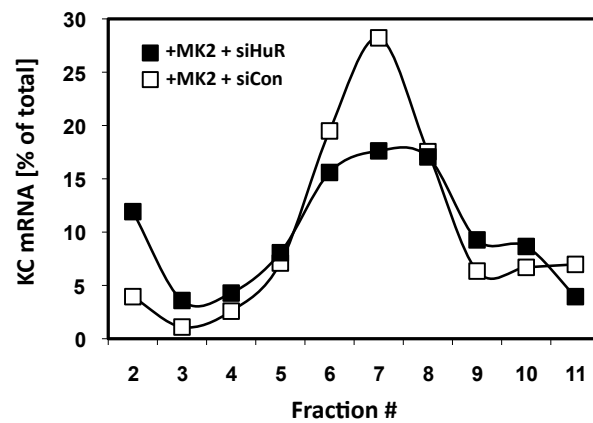

Supplement: Figure S8 — MK2-dependent stability and HuR-independent translation of KC mRNA. A) KC mRNA decay after 1 h of LPS treatment and subsequent inhibition of transcription using 5 µg/ml actinomycin D in MK2-rescued (+MK2) and GFP-transduced MK2/3-deficient immortalized macrophages (+GFP). mRNA levels immediately before addition of Act D were set to 100%. mRNA levels were determined by qRT-PCR before and at 30 and 60 min after Act D. Exponential regression analysis is shown. B) KC mRNA levels relative to actin mRNA in MK2-rescued (+MK2) and GFP-transduced MK2/3-deficient cells (+GFP) determined 1 h and 2 h after LPS stimulation. P values of the two-tailed student's t-test are given. C) Polysome profiling for KC mRNA in MK2-rescued cells upon HuR knockdown corresponding to Figure 4G and Figure S5. (PDF) [file pgen.1002977.s008.pdf]
